# Absorption Mode Broadband 2D MS for Proteomics and Metabolomics

**DOI:** 10.1021/jasms.6c00007

**Published:** 2026-04-27

**Authors:** Maria A. van Agthoven, Marek Polák, Jan Fiala, Claude Nelcy Ounounou, Petr Halada, Michael Palasser, Anne Briot-Dietsch, Alan Kádek, Kathrin Breuker, Petr Novák, Carlos Afonso, Marc-André Delsuc

**Affiliations:** † 27040 Université de Rouen-Normandie, Institut CARMeN UMR 6064, IRCOF, 1 rue Tesnière, 76821 Mont St Aignan Cedex, France; ‡ 86863 Institute of Microbiology, Czech Academy of Sciences, Videnska 1083, Prague 14220, Czech Republic; § Center for Chemistry and Biomedicine, 27255 University of Innsbruck, Innrain 80/82, 6020 Innsbruck, Austria; ∥ Faculty of Science, Charles University in Prague, Hlavova 6, Prague 12843, Czech Republic; ⊥ Institut de Génétique et de Biologie Moléculaire et Cellulaire, INSERM U596, CNRS UMR 7104, Université de Strasbourg, 1 rue Laurent Fries, 67404 Illkirch, Graffenstaden, France

**Keywords:** proteomics, metabolomics, tandem mass spectrometry, FT-ICR MS, two-dimensional mass spectrometry

## Abstract

Two-dimensional mass spectrometry (2D MS) is a method for tandem mass spectrometry that enables the correlation between precursor and fragment ions without the need for ion isolation. On a Fourier transform ion cyclotron resonance mass spectrometer, the phase correction functions for absorption mode data processing were found to be linear in the precursor ion dimension and quadratic in the fragment ion dimension. Phase-corrected absorption mode data processing on limited data sets has previously shown improvements in signal-to-noise ratio (SNR) and resolving power by a factor of 2. Here, we have expanded phase-corrected absorption mode data processing to 2D mass spectra regardless of size and frequency range. We have applied phase-corrected absorption mode 2D MS to top-down analysis of variously oxidized ubiquitin proteoforms generated by fast photochemical oxidation of proteins (FPOP) and to an extract of ergot alkaloids. We show that phase-corrected absorption mode data processing significantly improves both the SNR and the resolving power of the 2D mass spectrum compared to standard magnitude mode in terms of sequence coverage in top-down proteomics, as well as the accuracy of precursor-fragment correlation in metabolomics.

## Graphical abstract

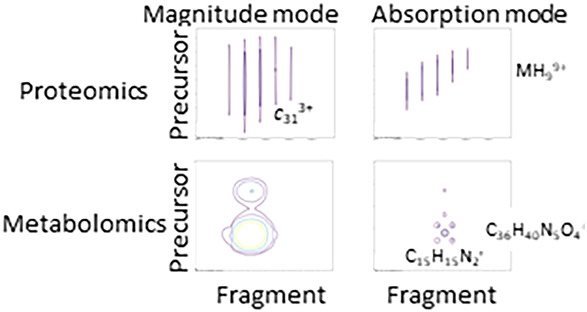

## Introduction

Two-dimensional mass spectrometry (2D MS) is a method for tandem mass spectrometry that enables the correlation between precursor and fragment ions without the need for ion isolation.^1^ On a Fourier transform ion cyclotron resonance mass spectrometer (FT-ICR MS), precursor ion radii in the ICR cell are modulated by using a pulse sequence developed by Pfändler *et al*. with an incremental delay.^2–4^ The modulation frequency is the cyclotron frequency of the ions minus an offset. Collision-free fragmentation methods in the ICR cell yield fragmentation efficiencies that are dependent on precursor ion radii.^5,6^ When precursor ion radii are modulated before fragmentation, fragment ion abundances are conversely modulated with the same frequency as their precursors, allowing for their correlation. A data set consists of transients recorded for each value of a series of regularly incremented delays. After Fourier transformation of each transient, a second Fourier transformation is performed according to incremental delay. The result is the 2D mass spectrum, which shows the fragmentation profiles of the ions from the analytes. To date, 2D MS has been performed on FT-ICR mass spectrometers with magnets between 7–15 T, with Infinity cells and with Paracells, and with a wide variety of fragmentation methods compatible with high vacuum in the ICR cell: infrared multiphoton dissociation (IRMPD), electron capture dissociation (ECD), electron-induced dissociation (EID), electron-detachment dissociation (EDD), or ultraviolet photodissociation (UVPD), demonstrating its applicability to a range of analytical scenarios.^7–10^

In one-dimensional FT-ICR MS, phase correction for absorption mode improves both the SNR and the resolving power of the mass spectrum.^11,12^ The phase of signals in the transient is found to be a quadratic function of cyclotron frequency.^13^ The zero-order, first-order, and second-order coefficients of the phase function have been found to depend mostly on instrument parameters.^14^ Multiple programs and algorithms have been developed to calculate phase correction functions and process mass spectra in phase-corrected absorption mode as easily as possible, although it has not yet been widely adopted by the FT-ICR community.^15,16^

For 2D MS, the phase correction function was found to be quadratic in the directly detected fragment ion frequency dimension and linear in the indirectly detected precursor ion frequency dimension, with the coefficients in the two dimensions independent from each other.^17^ Similarly to 1D MS, the first 2D mass spectrum to be processed in phase-corrected absorption mode showed improved SNR and resolving power in both dimensions.^18^ Crucially, phase-corrected absorption mode 2D MS was used to accurately correlate precursors and their fragments for a mixture of two histone peptides that differed only by *m*/*z* 0.006 (acetylation vs trimethylation), demonstrating the immense power hidden in this kind of analysis.^19^ However, unlike the automated magnitude mode data processing of 2D mass spectra, which relies on batch-processing, the Spectrometry Processing Innovative Kernel (SPIKE) software (the most widely used algorithm for 2D MS) required loading the entire data set in a computer’s random-access memory (RAM), thereby limiting the size of data sets that could be processed in phase-corrected absorption mode.^20^ Until now, phase-corrected absorption mode was only applied to narrowband mode 2D mass spectra, with precursor ion frequency ranges under 20 kHz.^21^ Most 2D mass spectra are acquired in broadband mode, with precursor ion frequency ranges between 150 kHz and 1 MHz (depending on magnetic field and the lowest precursor *m*/*z* ratio) to simultaneously fragment the whole mass range of analytes. Therefore, to be truly useful, phase correction for absorption mode data processing requires expansion to all frequency ranges and data sizes.

One of the current promising applications of 2D MS is top-down analysis of covalently modified biomolecules. Label-free relative quantification in 2D MS has enabled the identification, location, and quantification of modified peptides.^21^ Recently, 2D MS of chemically labeled ubiquitin tracked the solvent accessibility of its lysine residues.^22^ This result opens up the possibility of using 2D MS for the top-down analysis of proteins with other covalent-labeling techniques, such as Fast Photochemical Oxidation of Proteins (FPOP), in which hydroxyl radicals are generated with an excimer laser in a quench flow setup to oxidize solvent-accessible residues of proteins in a native-like environment.^23^ In most applications, oxidized proteins are digested before analysis by liquid chromatography coupled with tandem mass spectrometry (LC-MS/MS).^24^ Recently, FPOP has been combined with top-down analysis by direct infusion and tandem mass spectrometry on FT-ICR MS.^25–27^ The top-down analysis of FPOP-oxidized ubiquitin by 2D MS generated fragmentation patterns from multiple proteoforms, which enabled phase-correction for absorption mode processing.

Here, we address the limitation of phase-corrected absorption mode data processing to only narrowband mode 2D MS and RAM-limited data sizes with an update to the automated data processing program in SPIKE. We showcase its performance using a broadband 2D mass spectrum of FPOP oxidized ubiquitin and we compare the results to the same data set processed in magnitude mode. To demonstrate the wide applicability of this approach, we also performed phase-corrected absorption mode processing to a 2D UVPD mass spectrum of an extract of ergot alkaloids to examine the performance improvement of phase-corrected absorption mode for 2D MS in both top-down proteomics and metabolomics.

## Experimental Methods

### Sample Preparation

Ubiquitin from bovine erythrocytes was purchased from Sigma-Aldrich (Saint-Louis, MO, USA) and oxidized using the FPOP setup described by Yassaghi *et al.* ^25^ The solution was diluted to a 1 μM final protein concentration in aqueous solution of 1% acetic acid and 50% methanol for analysis. The extract of ergot alkaloids was isolated from *Claviceps (purpurea)* by HPLC and prepared in a 1:10 vol. solution of methanol.^28^ All solvents were LC-MS grade and obtained from Merck, Darmstadt, Germany.

### Instrumental Parameters

All 2D MS experiments were performed on a 15 T solariX FT-ICR mass spectrometer (Bruker Daltonik, Bremen, Germany) with an electrospray ion source operated in positive mode and direct infusion at a flow rate of 2 μL/min.

The pulse sequence for the 2D MS experiment is shown in Scheme 1. For ECD fragmentation, the two pulses in the encoding sequence (precursor detection and modulation) were set at 5.02 dB attenuation with 1.0 μs per excitation frequency step (frequency decrements were 625 Hz). The corresponding amplitude was estimated at 250 V_pp_, with a 1.9% sweep excitation power for an amplifier with a maximum output of 446 V_pp_. For UVPD fragmentation, the two pulses were set at 15.86 dB attenuation (estimated amplitude of 72 V_pp_, with an 0.4% sweep excitation) after optimization for the UVPD fragmentation zone in the ICR cell. In the horizontal fragment ion dimension, the excitation pulse in the detection sequence for all 2D mass spectra was set at 3.09 dB attenuation with a 20 μs/frequency step (frequency decrements were 625 Hz). The corresponding amplitude was estimated at 312 V_pp_, with a 35% sweep excitation power for an amplifier with a maximum output of 446 V_pp_.

**Scheme 1:**
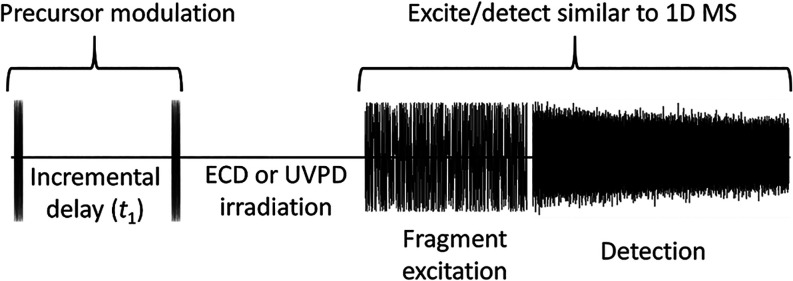
Pulse Sequence of the 2D MS Experiment

For the 2D mass spectrum of oxidized ubiquitin, ions were accumulated for 0.1 s before being transferred to the dynamically harmonized ICR cell (Paracell). The encoding delay *t*_1_ was increased 4096 times with a 2 μs increment, which corresponds to a 250 kHz frequency range. No phase-cycled signal averaging was employed in the experiment. The minimum cyclotron frequency for the modulated precursor ions was 92.2 kHz for a maximum *m*/*z* of 2500 during excitation, leading to a *m*/*z* 673.31–2500 mass range for precursor ions. Captured ions were fragmented by ECD. The hollow cathode current was 1.5 A. The ECD pulse length was set at 15 ms. The ECD lens was set at 25 V. The ECD bias was set at 1.5 V.^29^ The horizontal fragment mass range was *m*/*z* 207.274–2500 (corresponding to a frequency range of 1111.1–92.1 kHz). Transients were acquired over 0.236 s with 512k data points. The total duration of the experiment was 40 min.

For the 2D mass spectrum of the ergot alkaloid extract, continuous accumulation of selected ions (CASI) was used with two windows: *m*/*z* 550 (Δ*m*/*z* 100) and *m*/*z* 810 (Δ*m*/*z* 380) with 50 ms accumulation for each window to partially suppress the prominent base peak at *m*/*z* 610.^30^ The encoding delay *t*_1_ was increased 4096 times with a 2 μs increment, which corresponds to a 250 kHz frequency range. No phase-cycling or signal averaging was employed in the experiment. The minimum cyclotron frequency for the modulated precursor ions was 230.3 kHz for a maximum *m*/*z* of 1000 during excitation, leading to a *m*/*z* 479.52–1000 mass range for precursor ions. Captured ions were fragmented by a single 5 mJ pulse of a 193 nm excimer laser (Excistar 500, Coherent, Saxonbourg, USA), custom-coupled to the mass spectrometer through a BaF_2_ window at the rear of the ICR cell. The horizontal mass range was *m*/*z* 115.15–1000 (corresponding to a frequency range of 2000.0–230.3 kHz). Transients were acquired over 0.131 s with 512k data points. The total duration of the experiment was 38 min.

### Data Processing

The two-dimensional mass spectra were processed and visualized using SPIKE (available at www.github.com/spike-project, version 0.99.33, accessed on July 1, 2024) developed by the University of Strasbourg (Strasbourg, France) and CASC4DE (Illkirch-Graffenstaden, France) in the 64-bit Python 3.10 programming language on an open-source platform distributed by the Python Software Foundation (Beaverton, OR, USA).^20,31^ Processed data files were saved using the HDF5 file format.

The 2D mass spectra were processed in both magnitude and phase-corrected absorption mode. In both cases, batch processing was used to avoid loading the entire data set in RAM. Magnitude mode processing has been described previously.^32^ For phase-corrected absorption mode processing, each transient was apodized with a variant on the asymmetric apodization function proposed by Kilgour and Van Orden for phase-corrected absorption mode FT-ICR MS without baseline correction.^33^ Then, a quadratic phase correction function was applied to the frequency-domain spectrum of each transient. The coefficients of the phase correction function were optimized on a one-dimensional mass spectrum with the same experimental conditions for the excite-detect part of the pulse sequence. In the vertical precursor ion dimension, we chose a precursor ion scan with multiple peaks. We then performed linear phase correction for these peaks and applied it to all vertical precursor ion scans.^17,18^ The baseline of each precursor ion scan was corrected by recalculating the initial data points with an autoregressive model discussed in the results.

Processing of the ergot alkaloid sample was performed with Support Selection for Noise Elimination (SANE) denoising with the same apodization and zerofilling for visual purposes only (Figure 5a).^31,34^ In the rest of the study, no denoising algorithm is used. The program used for data processing in magnitude mode is available in SPIKE.^20^ The program used for data processing in phase-corrected absorption mode is available with the raw data set, alongside the configuration files for both magnitude mode and phase-corrected absorption mode processing of the 2D mass spectrum. In phase-corrected absorption mode, both processed 2D mass spectra had 8192 by 1 M data points. In magnitude mode, the 2D mass spectrum of oxidized ubiquitin had 8192 by 1 M data points and the 2D mass spectrum of ergot alkaloids had 8192 by 512k data points.

On a desktop computer with 128 GB of RAM and no message passing interface, processing of the 2D mass spectrum of ubiquitin took 2 h and 18 min in magnitude mode and 11 h and 31 min in absorption mode. The 2D mass spectrum of the ergot alkaloids was processed in 1 h and 3 min in magnitude mode and in 11 h and 17 min in absorption mode.

### Data Analysis

For each precursor ion species recorded in the 2D mass spectrum of oxidized ubiquitin, five fragment ion scans were added up to cover the precursor isotopic distribution and obtain isotopic distributions for all fragment ions as has been described in a previous study.^22^ The resulting one-dimensional fragment ion patterns were peak-picked in SPIKE. Frequency-to-mass conversion was quadratic in the horizontal fragment ion dimension.^35^ Peak assignments were performed using the Free Analysis Software for Top-down Mass Spectrometry (FAST-MS) developed by the University of Innsbruck (Innsbruck, Austria) in the 64-bit Python 3.7 programming language.^36,37^ FAST-MS generated theoretical *c*/*z* and *a* ^•^/*y* fragment lists for ubiquitin variably modified with 1–2 oxidations located on the following residues: phenylalanine, histidine, isoleucine, lysine, leucine, methionine, proline, arginine, valine, threonine, and tyrosine.^26,38^

For the 2D mass spectrum of the ergot alkaloid extract; peak-picking and centroiding were performed with a two-dimensional peak-picker in SPIKE and quadratic frequency-to-mass conversion was performed on the fragment ion scan of ergocryptine (*m*/*z* 576.3) and applied to all fragment ion scans and precursor ion scans.

All noise levels reported in this study were calculated by averaging the absolute value of the intensity over an empty segment of the spectrum close to the considered peak. The SNR was then calculated by dividing the intensity of the considered peak by the noise level.

## Results and Discussion

Top-down analysis of a mixture of proteoforms provides a germane test case for phase corrected absorption mode broadband 2D MS. To estimate the coefficients of the quadratic phase correction function in the horizontal dimension, a one-dimensional MS/MS spectrum with the same excite/detect parameters as the 2D MS pulse sequence (see Scheme 1) can be used, as shown in previous studies.^17–19^ Because in top-down analysis multiple charge states of very similar analytes are fragmented, a common fragment originating from enough precursors with a wide *m*/*z* range to estimate the coefficients of the linear phase correction function in the vertical dimension is easily found.^39^ The coefficients used for phase correction in absorption mode in 2D MS are listed in the configuration file for data processing. In applications with a wider diversity of analytes, where fragment ions present fewer precursor ion peaks in the 2D mass spectrum, an alternative strategy to estimate the coefficients of the linear phase correction function may be to sum multiple precursor ion scans prior to phase correction. Since the phase of fragment ion signals evolves the same way according to *t*_1_ (see Scheme 1) regardless of the fragment, we can hypothesize that this strategy enables the accurate estimation of the phase correction coefficients.^17^

In Figure 1, we show some of the fragmentation patterns of the 9+ charge state of ubiquitin after FPOP in the 2D mass spectrum after magnitude mode processing (Figure 1a) and phase-corrected absorption mode processing (Figure 1b). The fragmentation patterns of charge states ranging from 10+ to 6+ can be seen in Figure S1 in the Supporting Information. Both data processing methods show that linear phase correction is accurate in broadband mode over a frequency range of 250 kHz, which had only been adequately shown in narrowband mode with a 10 kHz frequency range.^18,19^ Increasing the frequency range leads to a steeper slope for the phase correction function. The accuracy with which the slope has to be determined, however, is unchanged. The number of data points in the vertical precursor dimension does not increase in a commensurate way with the frequency range and the number of peaks in precursor ion scans is also limited. These factors provide the main challenge in the progression from phase-correcting narrowband to broadband 2D MS.

**Figure 1:**
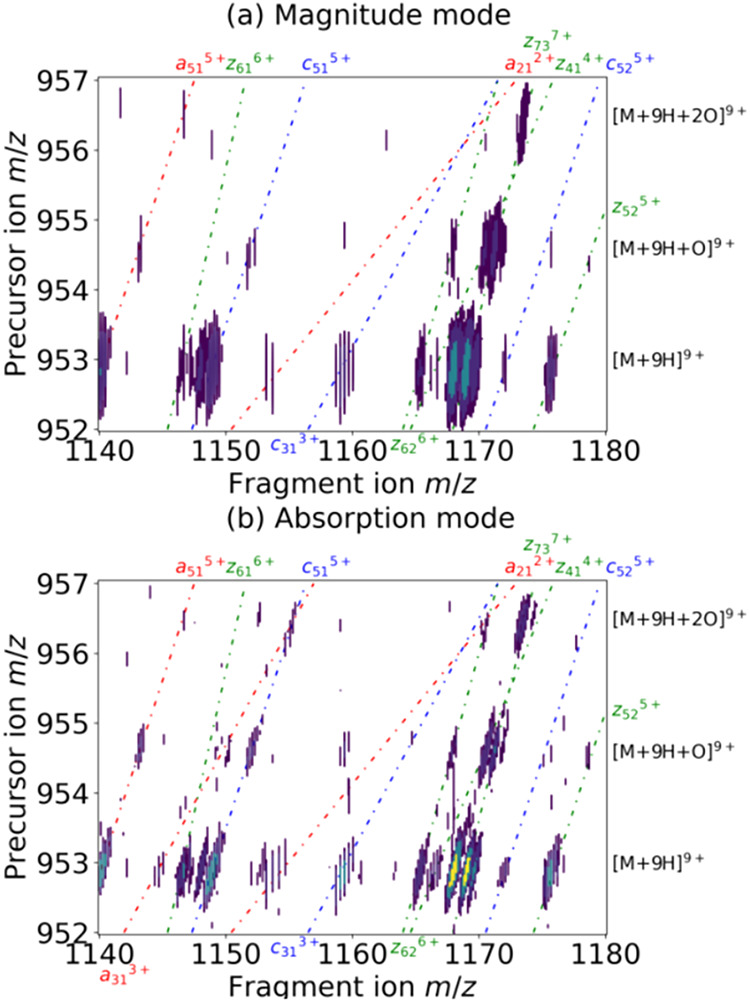
2D ECD mass spectrum of oxidized ubiquitin after (a) magnitude mode processing and (b) phase-corrected absorption mode processing. Dissociation lines are shown in dotted lines (red: *a*-type fragmentations, blue: *c*-type fragmentations, green: *z*-type fragmentations). For clarity, only a zoomed-in area of the 2D mass spectrum is presented. Areas of the 2D mass spectra with the fragmentation patterns of multiple charge states are shown in the Supporting Information (Figure S1).

2D MS experiments in previous studies using phase-corrected absorption mode were also limited in size because the entire file had to be loaded in the RAM for data processing. The program for phase-corrected absorption mode processing in this study was modified so that it only requires loading in RAM of a single row or column of the data set at a time, which essentially removes this limit.

Figure 1a,b are both contour plots. The lowest contour level is set slightly above noise level. Both figures show fragment ion peaks for the unmodified ubiquitin as well as ubiquitin bearing either one or two oxidations. Both the resolving power of the peaks and their SNR are significantly higher in phase-corrected absorption mode than in magnitude mode, as predicted. In the horizontal dimension, the resolving power at *m*/*z* 390 was measured to increase from 79,000 in magnitude mode to 188,000 in phase-corrected absorption mode. The resolving power at *m*/*z* 857 in the vertical dimension was measured to be 1,200 in magnitude mode and 2,900 in phase-corrected absorption mode. For the M+3 isotopic peak of *z*_73_^7+^ (mostly containing 2 ^13^C isotopes) at *m*/*z* 1168.8477 from the [M+9H]^9+^ precursor at *m*/*z* 952.69, the SNR was measured to be 24 horizontally and 16 vertically in magnitude mode. For the same peak in phase-corrected absorption mode, the SNR was 80 horizontally and 43 vertically. Because the data is hypercomplex, the theoretical gain in resolving power and SNR is 2 with all other parameters remaining equal. Here, for each processing mode, optimal apodization functions were used. The comparison in resolving power and SNR between the two spectra is therefore due to both the difference between magnitude mode and phase-corrected absorption mode and the apodization.^1,40^ In each dimension, the resolving power and SNR was at least doubled.

The observed improvement in resolving power and SNR leads to more fragment ions being detected and assigned with improved confidence (e.g., *a*_31_^3+^, *a*_21_^2+^for [M+9H]^9+^, and (*a*_51_+O)^5+^, (*c*_51_+O)^5+^, (*z*_62_+O)^6+^ for the [M+9H+O]^9+^ precursor in Figure 1). For comparison, a recent publication by Rahman *et al*. sought to improve the SNR of a 2D EDD mass spectrum of oligonucleotides by accumulating 8 scans for each value of *t*_1_ instead of 1 scan, with magnitude mode data processing.^10^ The theoretical improvement in SNR for accumulating 8 scans is approximately 2.8. This method multiplied the experiment time and sample consumption by a factor of 8 and improved the SNR by a factor of 1.3–3.0 without any improvement in resolving power.

One issue in phase-corrected absorption mode FT-ICR MS is the baseline of the spectrum. Because of the rapid rate of phase accumulation, any error at the start of the transient is amplified, which can lead to baseline oscillation in the vicinity of high intensity peaks and negative intensity peaks for ions of low abundance, which makes them invisible for peak-picking algorithms. Baseline oscillation also occurs in 2D mass spectra, as can be seen in phase-corrected absorption mode narrowband 2D mass spectra.^18,19^ One solution to this problem is to perform postprocessing baseline correction. However, we sought to avoid excessive data manipulation after processing. A less computational time-consuming solution, is the use of an asymmetrical apodization function proposed by Kilgour and Van Orden, which sets the initial data points of transients at zero.^33^ Their proposed apodization function A has a rise and fall with a maximum at a fraction F of the transient length. Its equation is

$$\mathrm{For}\,\;\;n\leq \mathrm{NF}:A\left(n\right)=\frac{1}{2}\left(1-\cos\left(\frac{n\pi }{\mathrm{NF}-1}\right)\right)$$ 1

$$\mathrm{For}\,\;\;n>\mathrm{NF}:A\left(n\right)=\frac{1}{2}\left(1-\cos\left(\frac{\left(n+N\left(1-F\right)-1\right)\pi }{N\left(1-F\right)-1}\right)\right)$$ 2

in which *N* is the number of data points in the transient, *F* the fraction of transient at which the apodization function is at its maximum, *n* the *n*th data point in the transient.

Here, we used a variant of the Kilgour-Van Orden apodization function. For *n* > *NF*, we used $\sqrt{\left|A\left(n\right)\right|}$ instead of the function in eq 2. We show this function in Figure 2a with a maximum at *F* = 0.25, which we found to be optimal for our baseline correction and SNR.

**Figure 2:**
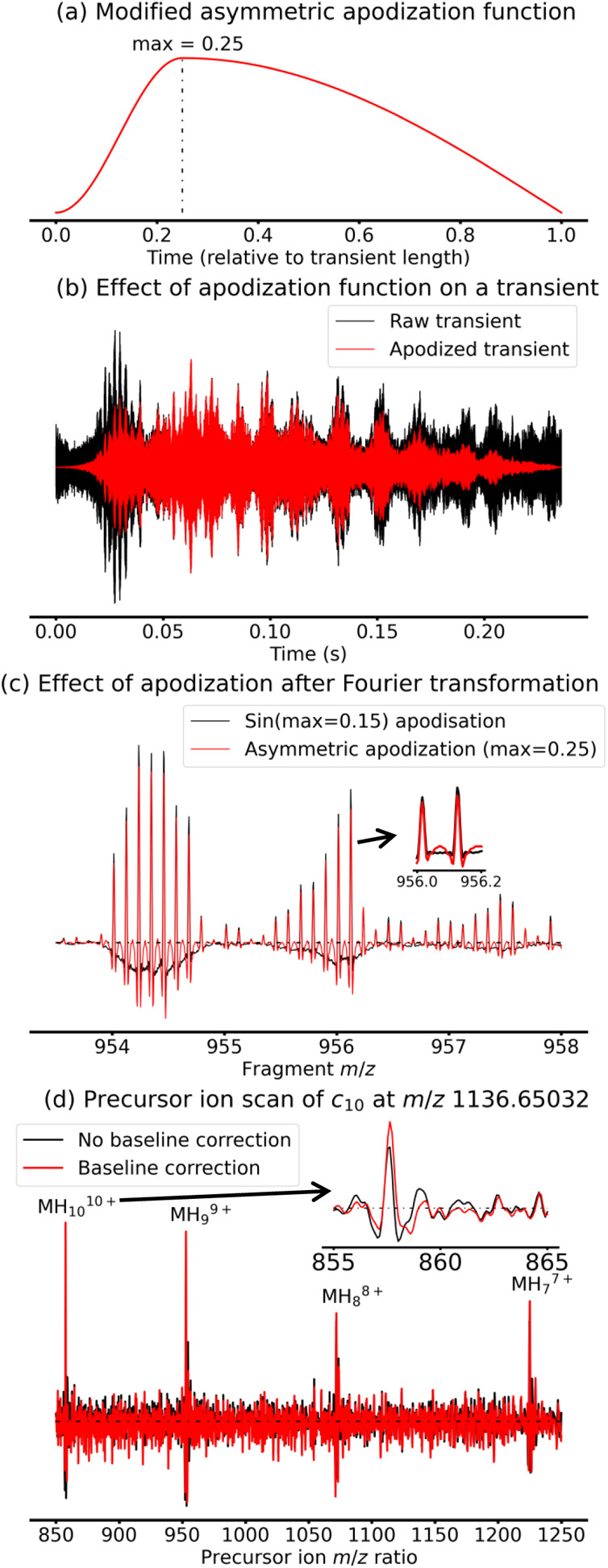
(a) Profile of the modified asymmetric apodization function proposed by Kilgour and Van Orden.^33^ (b) Transient extracted from the 2D mass spectrum at *t*_1_ = 1126 μs without apodization (black) and with the apodization from Figure 2a (red). (c) Comparison between the phase-corrected absorption mode mass spectrum at *t*_1_ = 1126 μs obtained with sinusoidal apodization (black) and the asymmetric apodization shown in Figure 2a (red). (d) Precursor ion scan at *m*/*z* 1136.65032 without baseline correction (black) and with baseline correction (red). In (c–d) the dotted black line indicates zero intensity.

Figure 2b explains our choice of apodization function by showing the effect of apodization (red) on a randomly chosen transient (black) from the 2D MS data set. The start of the transient is suppressed by the apodization to correct the baseline using eq 1. To limit the duration of 2D MS experiments, shorter transients are preferred during data acquisition. Hence, at the end of the transient, the decay of the signal is often cut short by the end of the acquisition. Therefore, we chose to apodize the transient with the square root of the asymmetric function in eq 2 to minimize signal loss at the transient end.

The result of the Fourier transformation in phase-corrected absorption mode (Figure 2c, red) shows a corrected baseline compared to the same data processing with a standard shifted Sine Bell apodization (Figure 2c, black). As a side note, the peaks, which represent the [M+9H]^9+^ precursors and their isoforms, form abnormal isotopic distributions due to radius modulation (see Scheme 1). The peaks also show intense side-lobes that are similar to the ones presented by Kilgour and Van Orden, but which do not hinder data analysis, since they are negative.^33^

The signal in the vertical dimension also has a rapid rate of phase accumulation which cannot be corrected as easily as the transients. Instead, we chose to use an autoregressive model to correct errors in the initial data points. This model is based on the fact that, for a data set with *N* data points, each real data point *x*(*n*) at detection date or index *n* can be expressed as the sum of the signal from the sample *x̂*(*n*) and the noise *e*(*n*):

3 x(n)=x̂(n)+e(n)

In 2D MS, the signal *x̂*(*n*) can be expressed as a finite set of regularly sampled, exponentially dampened sinusoids:^41^

4 $$\mathrm{x̂}\left(n\right)=\sum_{k=1}^{p}\alpha_{k}{\left(z_{k}\right)}^{n}$$

where α_*k*_ are complex amplitudes (containing the phase information) and *z*_*k*_ are the poles of the signal and *p* is the number of poles:

5 $$z_{k}=e^{\gamma_{k}+j\omega_{k}}$$

where ω_*k*_ are the frequencies of the signal and γ_*k*_ the dampening factors. The signal can therefore be decomposed in *p* [α_*k*_, *z*_*k*_] vectors and is predictable. If *N* > *p*, then the value of *x̂*(*n*) can be predicted for *p* < *n* ≤ *N*. Conversely, the data points between *N–p* and *N* can be used to calculate the data points for 1 ≤ *n* ≤ *N*–*p*–1.

In the vertical dimension of 2D mass spectra, *p* corresponds approximately to the number of peaks. (i.e., the number of precursors a given fragment has). Here, the phase was found to rotate 392 times over the signal for the highest frequencies (see Supporting Information, configuration file for phase-corrected absorption mode data processing).^18^ Since the phase in the vertical dimension accrues linearly, we estimated phase rotations started over the 392 first points of each vertical precursor ion scan and that they should be corrected. We plotted the result in Figure 2d (red) for the precursor ion scan of the *c*_10_ fragment and compared it to the precursor ion scan before baseline correction (black). As can be seen on Figure 2d, especially for the peak corresponding to the [M+10H]^10+^ precursor, baseline correction somewhat suppresses the negative side-lobes, but, more importantly, suppresses the positive side-lobes which can be misinterpreted in the 2D mass spectrum (although it should be noted that artifacts are not affected by baseline correction). In addition, the apparent SNR for [M+10H]^10+^ increased from 15 to 20 with baseline correction, because the baseline fluctuation before correction was mistaken for noise.

The improved resolving power and SNR in phase-corrected absorption mode 2D MS leads to higher confidence in peak assignment. In Figure 3a, we show the fragmentation pattern of 10+ ubiquitin extracted from the 2D mass spectrum after data processing in magnitude mode (black) and in phase-corrected absorption mode (red). To show complete fragment ion isotopic distributions of [M+10H]^10+^, we summed up 5 fragment ion scans between precursor ion *m*/*z* 857.3–857.7 to construct the spectra shown in Figure 3a. Peak assignment was performed in FAST-MS without any modifications (all fragment assignments for these peaklists are reported in Tables S1 and S2 in the Supporting Information).^37^ In FAST-MS, the false positive discovery rate is evaluated at 5%. In this study, all peak assignments were manually inspected. In Figure 3a, peaks assigned in both magnitude and phase-corrected absorption mode are labeled in black, and those only assigned in phase-corrected absorption mode are labeled in red. Between fragment *m*/*z* 1100–1140 alone, 3 more fragments were assigned in phase-corrected absorption mode (*c*_20_^2+^, *z*_50_^5+^, and *z*_70_^7+^). After manual inspection of the automatic peak assignments, we found that the sequence coverage was 92% in phase-corrected absorption mode with 193 assigned fragments compared to 82% in magnitude mode with 146 assigned fragments (detailed peak assignment tables and sequence coverage in SI Tables S1 and S2 and Schemes S1 and S2).

**Figure 3:**
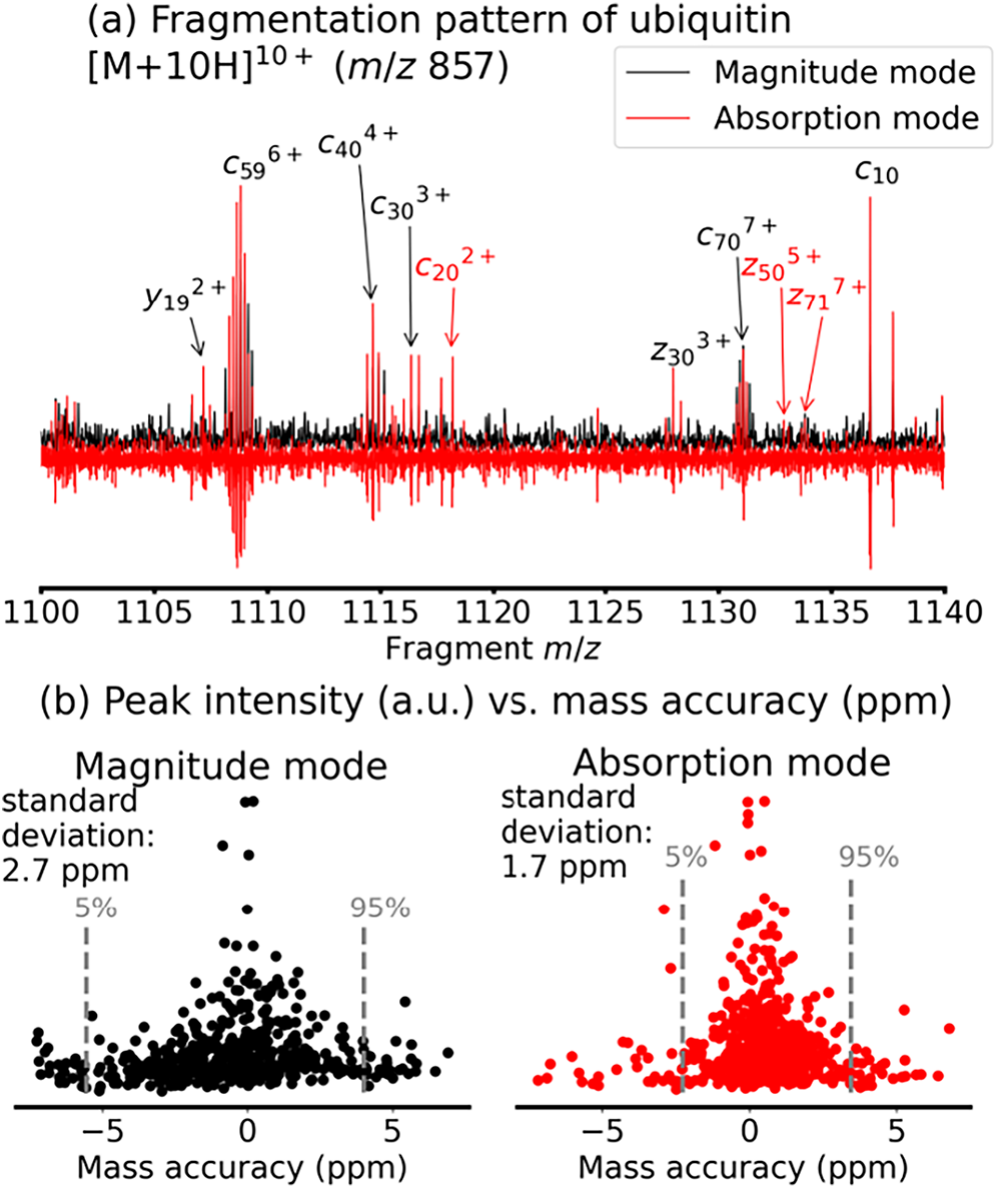
(a) Fragmentation pattern of ubiquitin [M+10H]^10+^ obtained by the sum of fragment ion scans between precursor *m*/*z* 857.3–857.7 in magnitude mode (black) and phase-corrected absorption mode (red). The peak assignments in black were found in both magnitude mode and phase-corrected absorption mode. The peak assignments in red were found only in phase-corrected absorption mode. (b) Normalized peak intensity vs mass accuracy for assigned peaks in magnitude mode (left) and phase-corrected absorption mode (right).

Mass accuracy reflects the confidence of peak assignments, and therefore we plotted normalized peak intensities vs mass accuracy in Figure 3c. In phase-corrected absorption mode (right), mass errors cluster closer to 0 ppm than in magnitude mode (left) with a smaller standard deviation (1.7 vs 2.7 ppm), thereby improving confidence in peak assignments. The phase-corrected absorption mode-induced increase of ion assignments observed for unmodified ubiquitin also applies to the fragments of oxidized ubiquitin, which thereby increases the structural resolution in mapping ubiquitin solvent accessibility.

We extracted fragment ion scans from the phase-corrected absorption mode 2D mass spectrum to construct the complete isotopic distribution of the fragmentation patterns of oxidized ubiquitin before peak-picking and peak assignment. Because the oxidation sites of ubiquitin in FPOP are *a priori* unknown, using externally calibrated peaklists for peak assignments can cause errors. For example, *y_n_* and *z_n_* +ox fragments are isobaric and get more difficult to distinguish when their charge state increases. In 2D MS, the internal calibration for quadratic frequency-to-mass conversion of one fragment ion scan can be applied to all the others for high mass accuracy. Here, we applied the internal calibration of the 9+ charge state of unmodified ubiquitin to all the other fragment ion scans. FAST-MS performed peak assignments on the calibrated peaklists of ubiquitin with 1 or 2 oxidations at all 4 charge states that were detected in the 2D mass spectrum (10+–7+).^37^ Oxidations were enabled at the following residues: F, H, I, K, L, M, P, R, V, W, Y.^23^ As can be seen in the assignment examples shown in Figure 4a, FAST-MS generated theoretical distributions that can be compared to peak *m*/*z* ratios and intensities for assignments.

**Figure 4:**
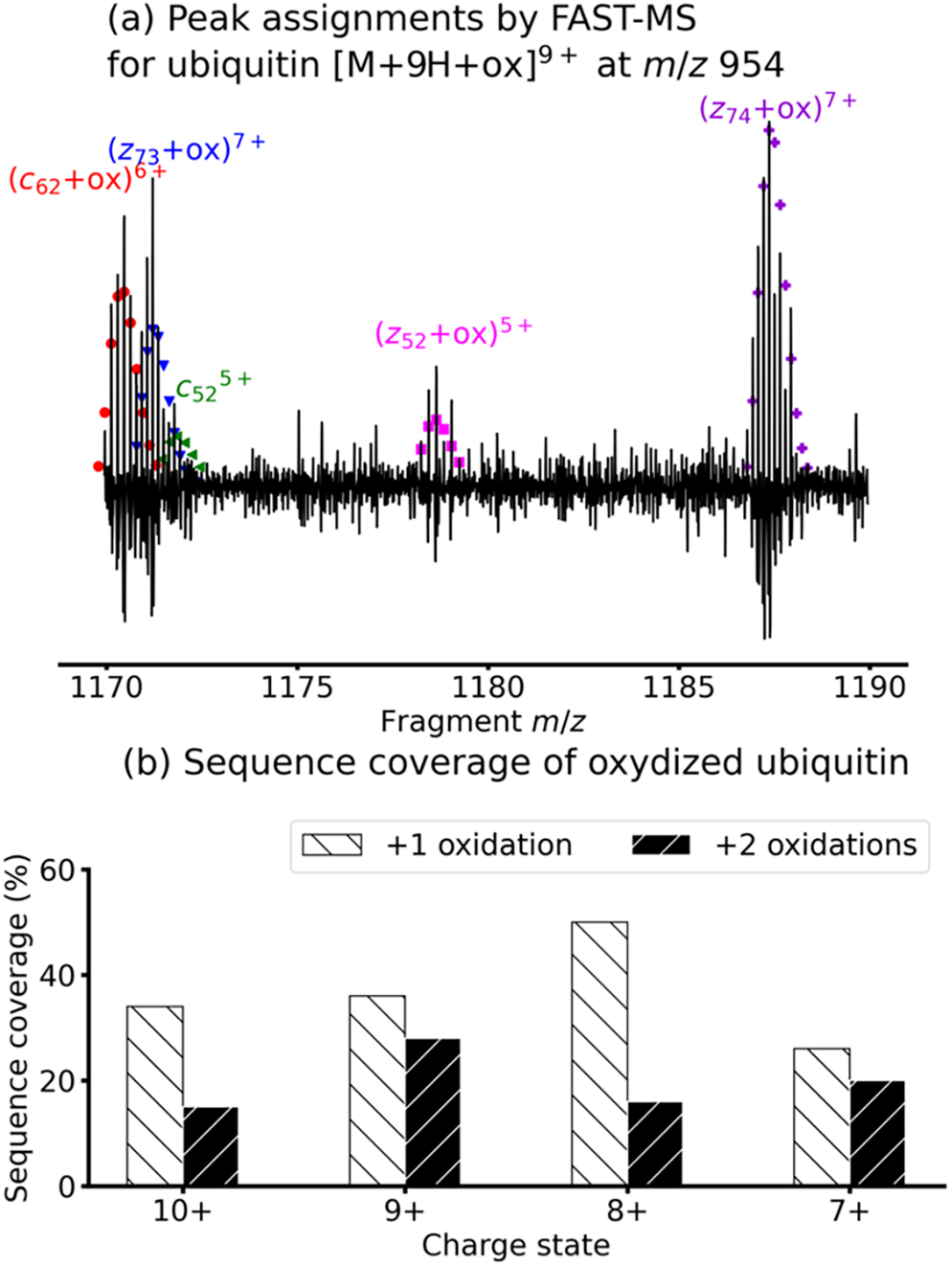
Data analysis of the 2D mass spectrum in phase-corrected absorption mode. (a) Extracted fragment ion scan of [M+9H+ox]^9+^ at *m*/*z* 954 and peak assignments by FAST-MS (colored dots). (b) Sequence coverage of oxidized ubiquitin after peak assignment by FAST-MS for the extracted fragment ion scans after reconstruction of the isotopic distribution for ubiquitin with 1 and 2 oxidations for all charge states in the 2D mass spectrum.

As can be seen with the assignments of the isotopic distribution of (*c*_62_+ox)^6+^, (*z*_73_+ox)^7+^, and *c*_52_^5+^, FAST-MS is capable of making assignments of overlapping isotopic distributions. Because we chose to sum up 5 fragment ion scans for each precursor ion, we were not able to recover the full isotopic distributions of each fragment ion, hence the discrepancies observed between the theoretical and the experimental isotopic distributions in Figure 4a.

A one-dimensional mass spectrum of the oxidized ubiquitin (see Figure S2) was deconvolved in the DataAnalysis 6.0 software (Bruker Daltonics, Bremen, Germany) using the Sophisticated Numerical Annotation Procedure (SNAP) algorithm.^42^ The overall extent of oxidation was determined to be 40% for one oxidation and 28% for two oxidations. As a result, fragment ion abundances and therefore SNR are lower for oxidized ubiquitin species, since they are dependent on precursor ion abundances, i.e., amount of incorporated oxygen. For ubiquitin with one oxidation, the sequence coverage was found to be between 26 and 50% according to charge state, as shown in Figure 4b. Similarly, for ubiquitin with 2 oxidations, the sequence coverage was found to be between 15 and 28% (peaklists are available in the Supporting Information). For 2D MS to compete with tandem mass spectrometry for top-down FPOP analysis, isolating one charge state of ubiquitin proteoforms, increasing accumulation times and performing narrowband 2D MS would be preferable to broadband 2D MS.^21^

Top-down proteomics is not the only application in which analytes with chemical similarities yield fragment ions with the same *m*/*z*. Mass spectra of samples in metabolomics and the analysis of natural compounds tend to also be complex with many peaks close in mass and chemical composition, which can produce precursor ion scans with multiple peaks in 2D MS. This property makes them eminently suitable for broadband phase-corrected absorption mode 2D MS. A convenient example for testing our algorithm is the 2D UVPD mass spectrum of an extract of ergot alkaloids, shown in Figure 5. Ergot alkaloids are found as fungal growth-induced contamination in cereal products and may have toxic (e.g., ergotism) or pharmacologically beneficial effects upon ingestion.^43^ They are composed of an ergoline ring that is methylated on the N-6 nitrogen and substituted with a peptidic ring on C-8.^44^

**Figure 5:**
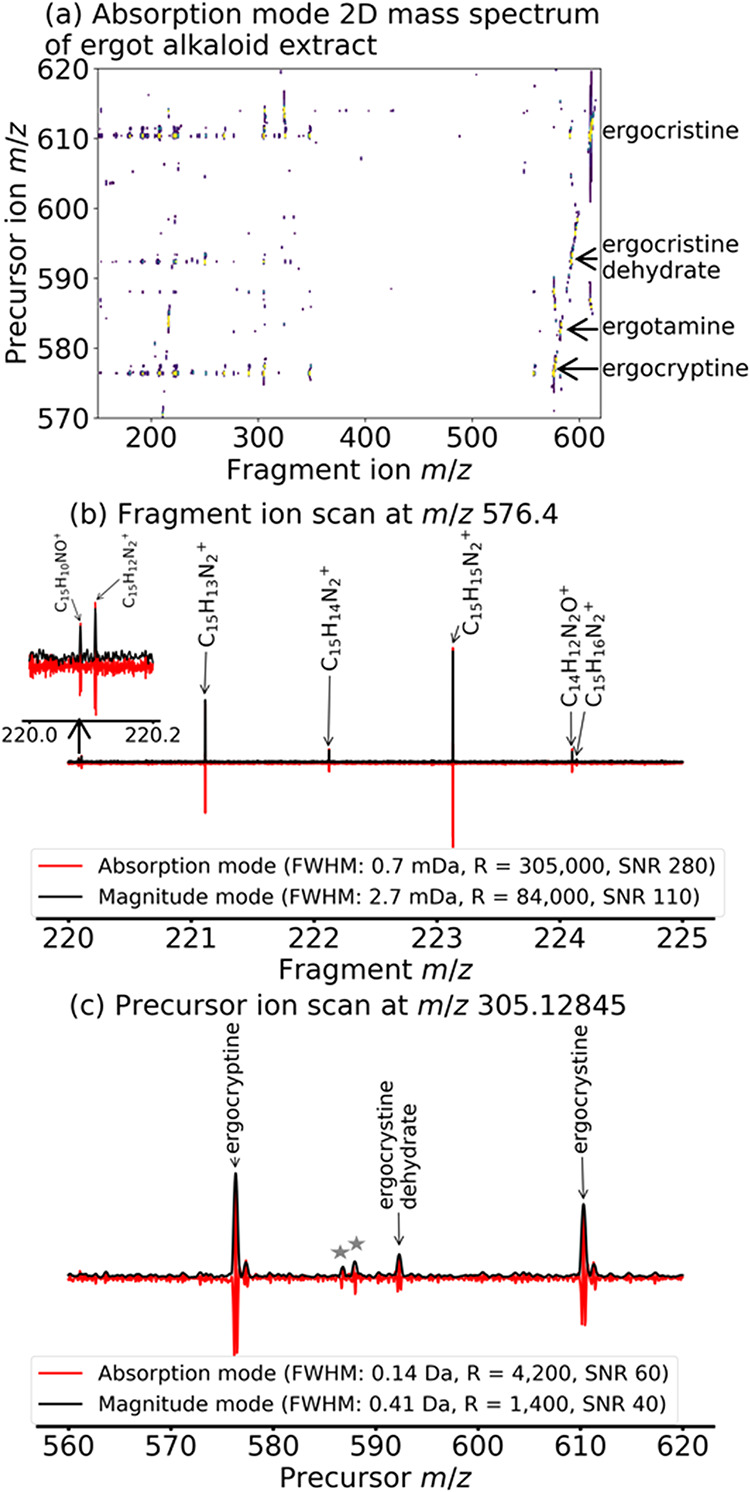
(a) Phase-corrected absorption mode 2D UVPD mass spectrum of an extract of ergot alkaloids (with SANE denoising). (b) Extracted fragment ion scan of ergocryptine (C_32_H_42_N_5_O_5_^+^ at *m*/*z* 576.31805): comparison between absorption mode and magnitude mode (no denoising). (c) Extracted precursor ion scan of C_19_H_17_N_2_O_2_^+^ (*m*/*z* 305.12845): comparison between absorption mode and magnitude mode (no denoising).

To reduce the intensity of the ergocristine peak (C_35_H_40_N_5_O_5_^+^) at *m*/*z* 610.30240, which would otherwise have dominated the mass spectrum, CASI isolation was applied in the front-end of the instrument for controlled peak suppression.^30^ The mass spectrum of the ergot alkaloid extract (shown in full in Figure S4 in the Supporting Information) enabled the assignment of multiple structurally similar ergot alkaloids in the sample, such as ergocryptine, ergotamine, ergocristine, and ergocristine dehydrate (see peak assignments in Table S11 of the Supporting Information).^45^ All ions in the mass spectrum and 2D mass spectrum were singly charged. In addition to these known compounds, we assigned a peak at *m*/*z* 592.25542 to C_34_H_34_N_5_O_5_^+^.

From the same absorption mode-processed 2D mass spectrum shown in Figure 5a, we were able to extract the fragmentation patterns of ergocristine, ergocristine dehydrate, and ergocryptine, by relying on the fragmentation patterns obtained with collision-induced dissociation (CID) in previous studies by Lehner *et al*.^45^ The fragmentation patterns obtained in UVPD were very similar to the ones obtained in CID. All peak assignments were manually inspected. We used a precursor ion scan with multiple known peaks to calculate the coefficients of the linear phase correction function. Figure 5b shows the comparison between the fragment ion scan of ergocryptine extracted at *m*/*z* 576.4 in phase-corrected absorption mode and in magnitude mode. Measurements of the resolving power and the SNR were taken for the peak at *m*/*z* 223.12304. The magnitude mode 2D mass spectrum was processed with one zerofill horizontally compared to two zerofills in phase-corrected absorption mode, which explains the 3.6 factor improvement in resolving power instead of the factor of approximately 2 that is expected. The average mass error of peak assignments also decreased from an 0.35 ppm in magnitude mode to 0.10 ppm in phase-corrected absorption mode (see peak assignments in Tables S12 to S19 in the Supporting Information). In the precursor ion dimension, the resolving power and SNR both increase from magnitude mode to phase-corrected absorption mode (see Figure 5c) by a factor of 3 and 1.5 respectively.

Due to baseline isotopic resolution in the vertical precursor ion dimension, we were able to perform peak-picking and centroiding in two dimensions. Doing so allowed us to measure both precursor and the fragment *m*/*z* of all fragment peaks. For the fragments of ergocryptine (*m*/*z* 576.31804), the difference between maximum and minimum precursor *m*/*z* was 220 mDa in magnitude mode (standard deviation: 38 mDa) and 45 mDa in phase-corrected absorption mode (standard deviation: 6 mDa). For the fragments of ergocristine (*m*/*z* 610.30234), the range of precursor *m*/*z* measurements was 111 mDa in magnitude mode (standard deviation: 22 mDa) and 24 mDa in phase-corrected absorption mode (standard deviation: 3 mDa). These results are consistent with precursor *m*/*z* measurements reported by Marzullo *et al*.^8^

When checking against the autocorrelation line and the one-dimensional mass spectrum, the fragments at precursor *m*/*z* 592.3 have two possible precursors: C_35_H_38_N_5_O_4_^+^ at *m*/*z* 592.29182 (ergocristine dehydrate) and C_34_H_34_N_5_O_5_^+^ at *m*/*z* 592.25542 (unknown), which have a mass difference of 36 mDa (CH_4_ vs O), which would not be separable with standard quadrupolar precursor isolation. In Figure 6a, we plotted the normalized precursor ion scans of *m*/*z* 250.09748 (assigned to C_15_H_12_N_3_O^+^) and *m*/*z* 305.12845 (assigned to C_19_H_17_N_2_O_2_^+^). The former fragment only has one precursor at *m*/*z* 592.3 and the latter fragment has precursors at *m*/*z* 576.3, *m*/*z* 592.3, and *m*/*z* 610.3 (see Figure 5c). The noise level for the precursor ion scan of *m*/*z* 305.12845, resulting mainly from scintillation noise which is proportional to the intensity of the signal, is therefore higher than the noise level of the precursor ion scan of *m*/*z* 250.09748.^41^ In Figure 6a, the two peaks are shifted, suggesting that the precursor for *m*/*z* 250.09748 is C_34_H_34_N_5_O_5_^+^ and the precursor for *m*/*z* 305.12845 is C_35_H_38_N_5_O_4_^+^ (ergocristine dehydrate). However, the vertical resolving power in magnitude mode (R = 1300 at *m*/*z* 592.3) is not sufficient to confidently assign precursor ions. In contrast, after phase-corrected absorption mode processing, the vertical resolving power dramatically increases to 6200 at *m*/*z* 592.3, as plotted in Figure 6b. Here, the shift between the two peaks is more pronounced.

**Figure 6:**
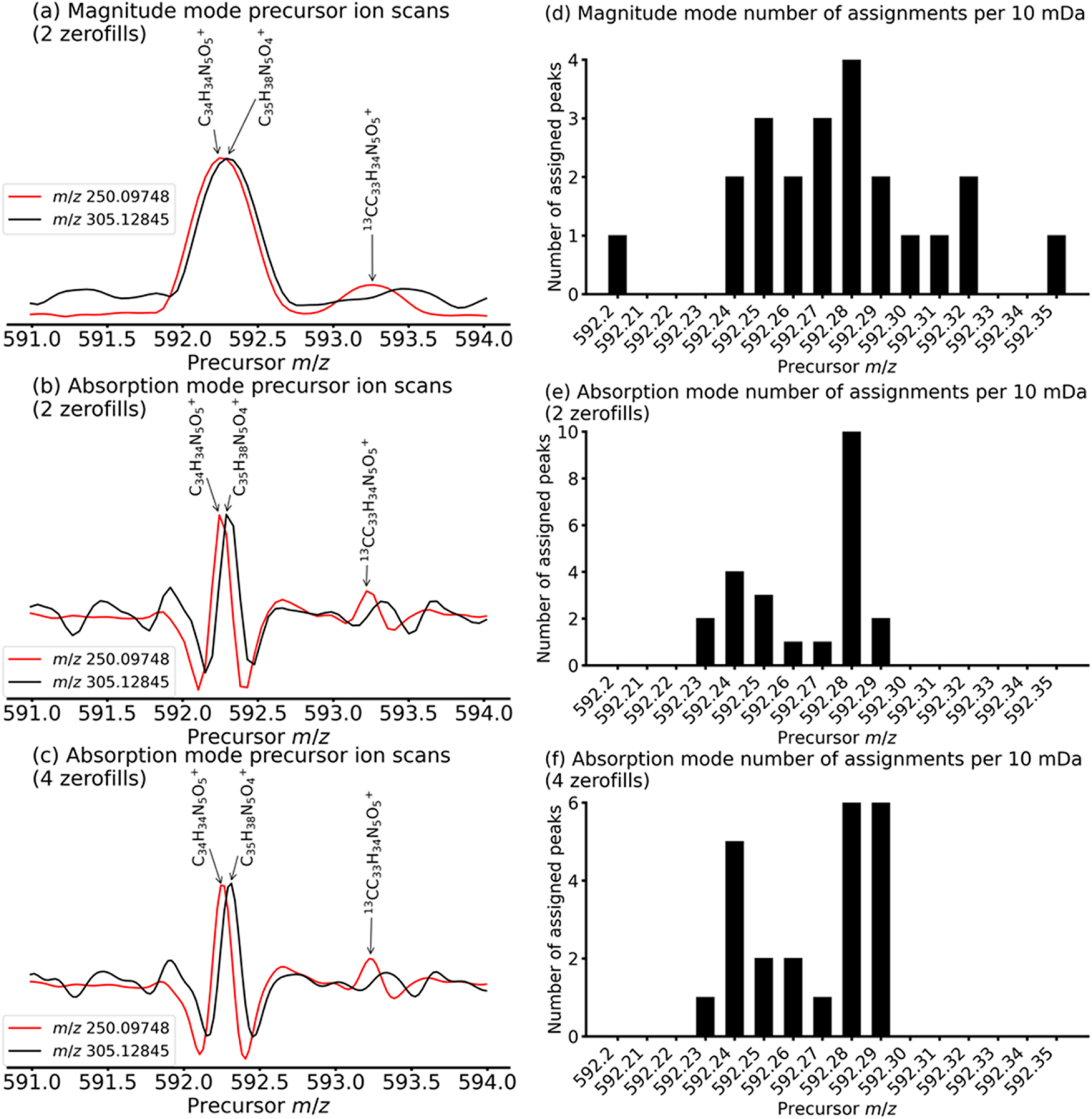
Comparison between the normalized precursor ion scans of the fragment at *m*/*z* 250.09748 (C_15_H_12_N_3_O^+^) and the fragment at *m*/*z* 305.12845 (C_19_H_17_N_2_O_2_^+^) extracted from the (a) magnitude mode, (b) phase-corrected absorption mode 2D mass spectrum of the ergot alkaloid extract after processing with 2 zerofills in the vertical precursor ion dimension, and (c) phase-corrected absorption mode 2D mass spectrum of the ergot alkaloid extract after processing with 4 zerofills in the vertical precursor ion dimension. Number of peak assignments per 10 mDa for peaks detected in the precursor *m*/*z* 592–593 and fragment *m*/*z* 120–600 region of the (d) magnitude mode, (e) phase-corrected absorption mode 2D mass spectrum of the ergot alkaloid extract (2 zerofills), and (f) phase-corrected absorption mode 2D mass spectrum of the ergot alkaloid extract (4 zerofills).

In Palasser *et al.*, accurate precursor-fragment correlation was shown to limited by the distance between adjacent data points in the vertical precursor dimension.^19^ In Figure 6b, the distance between data points in the vertical precursor dimension is 31 Hz in the frequency domain. At 15 T, the difference in cyclotron frequency between *m*/*z* 592.29182 and *m*/*z* 592.25542 is 24 Hz. The distance between data points is therefore not sufficient to confidently differentiate between the two precursors. After phase-corrected absorption mode processing with 4 zerofills instead of 2, the distance between data points in the vertical precursor dimension in the frequency domain is 15 Hz, which is sufficiently low to accurately correlate the fragments with each precursor, as can be seen in Figure 6c, where the distance between data points has been halved.

To further distinguish between the two precursor ions, we sorted the assigned fragment ion peaks in the precursor *m*/*z* 592.20–592.35 range in *m*/*z* 0.01 bins. In Figure 6d, the precursor *m*/*z* of the fragment ion peaks from the magnitude mode 2D mass spectrum are within an *m*/*z* 592.20–592.35 range and the two populations overlap. We therefore cannot distinguish between the fragments of C_35_H_38_N_5_O_4_^+^ and C_34_H_34_N_5_O_5_^+^. In Figure 6e, however, the precursor *m*/*z* of the fragment ion peaks from the phase-corrected absorption mode 2D mass spectrum are within an *m*/*z* 592.22–592.29 range. Two separate ion populations can be distinctly observed, and the fragments of C_35_H_38_N_5_O_4_^+^ and C_34_H_34_N_5_O_5_^+^ can therefore be clearly distinguished. This result is confirmed in Figure 6f, in which the precursor *m*/*z* are sorted for the 2D mass spectrum in phase-corrected absorption mode with 4 zerofills in the vertical precursor ion dimension. However, we see more fragments with precursor ion *m*/*z* 592.25–592.27. These fragments are likely common to both C_35_H_38_N_5_O_4_^+^ and C_34_H_34_N_5_O_5_^+^.

## Conclusion

Removing the limitation of data size, imposed by computational memory, by adopting batch-processing, enabled us to successfully phase-correct a broadband 2D mass spectrum of FPOP-oxidized ubiquitin in both dimensions for phase-corrected absorption mode, as a model top-down MS study of a protein with many proteoforms. We were also able to correct the baseline of the 2D mass spectrum in both the precursor and fragment ion dimension, with a variant on the Kilgour-Van Orden asymmetrical apodization, and in the precursor ion dimension, with an autoregressive denoising algorithm. When comparing the resulting phase-corrected absorption mode 2D mass spectrum with the magnitude mode 2D mass spectrum processed from the same data set, we demonstrate improved SNR, resolving power, mass accuracy and ultimately peak-assignment and protein sequence coverage.

We also applied our approach and phase-corrected absorption mode batch-processing to the 2D UVPD mass spectrum of an extract of ergot alkaloids. Due to their structural similarities, the compounds in the sample have common fragments whose signals can be used to calculate phase correction coefficients in the precursor ion dimension. Here we also observed increased resolving powers and SNR by a factor of 2–3. The increased resolving power in particular enabled the accurate correlation between precursor and fragment ions for two precursor ions with *m*/*z* differences as low as 36 mDa.

The optimization of the coefficients of the phase correction functions remains onerous and would gain from automation.^16^ In a future study, we will also investigate the reuse of processing parameters for multiple 2D mass spectra acquired in identical experimental conditions.^14^ However, already the two presented examples of very diverse applications clearly demonstrate how phase-corrected absorption mode data processing improves the performance of 2D MS, and the strengths that FT-ICR phasing and absorption mode processing offer for highly complex samples.

## Supplementary Material



## Notes

Peaklists with assignments and additional figures (PDF)
  Phase-corrected absorption mode data processing program (python program file). Data processing parameter files (text files). Raw data files (miscellaneous files). All associated contents available free of charge at: 10.5281/zenodo.18018080.

## #

ICR Program, National High Magnetic Fields Laboratory, 1800 East Paul Dirac Drive, Tallahassee FL32310, United States

## ∇

Thermo Fisher Scientific, Achtseweg Noord 5, 5651 GG Eindhoven, Netherlands.

## ○

Bachem AG, Hauptstrasse 144, 4416 Bubendorf, Switzerland.

## Abbreviations

2D MS: two-dimensional mass spectrometry
FPOP: fast photochemical oxidation of proteins
FT-ICR MS: Fourier transform ion cyclotron resonance mass spectrometry
ECD: electron capture dissociation
UVPD: ultraviolet photodissociation
SNR: signal-to-noise ratio

## References

[1] van
Agthoven M. A., Lam Y. P. Y., O’Connor P. B., Rolando C., Delsuc M.-A. (2019). Two-Dimensional Mass Spectrometry:
New Perspectives for Tandem Mass Spectrometry. Eur. Biophys. J..

[2] Pfändler P., Bodenhausen G., Rapin J., Houriet R., Gäumann T. (1987). Two-Dimensional
Fourier Transform Ion Cyclotron Resonance Mass Spectrometry. Chem. Phys. Lett..

[3] Pfaendler P., Bodenhausen G., Rapin J., Walser M. E., Gäumann T. (1988). Broad-Band
Two-Dimensional Fourier Transform Ion Cyclotron Resonance. J. Am. Chem. Soc..

[4] Bensimon M., Zhao G., Gäumann T. (1989). A Method to
Generate Phase Continuity
in Two-Dimensional Fourier Transform Ion Cyclotron Resonance Mass
Spectrometry. Chem. Phys. Lett..

[5] van
Agthoven M. A., Chiron L., Coutouly M.-A., Sehgal A. A., Pelupessy P., Delsuc M.-A., Rolando C. (2014). Optimization of the
Discrete Pulse Sequence for Two-Dimensional FT-ICR Mass Spectrometry
Using Infrared Multiphoton Dissociation. Int.
J. Mass Spectrom..

[6] van
Agthoven M. A., Lynch A. M., Morgan T. E., Wootton C. A., Lam Y. P. Y., Chiron L., Barrow M. P., Delsuc M.-A., O’Connor P. B. (2018). Can Two-Dimensional IR-ECD Mass Spectrometry Improve
Peptide de Novo Sequencing?. Anal. Chem..

[7] van
Agthoven M. A., Wootton C. A., Chiron L., Coutouly M.-A., Soulby A., Wei J., Barrow M. P., Delsuc M.-A., Rolando C., O’Connor P. B. (2016). Two-Dimensional Mass Spectrometry
for Proteomics, a Comparative Study with Cytochrome c. Anal. Chem..

[8] Marzullo B. P., Morgan T. E., Wootton C. A., Perry S. J., Saeed M., Barrow M. P., O’Connor P. B. (2020). Advantages
of Two-Dimensional Electron-Induced
Dissociation and Infrared Multiphoton Dissociation Mass Spectrometry
for the Analysis of Agrochemicals. Anal. Chem..

[9] Marzullo B. P., Morgan T. E., Theisen A., Haris A., Wootton C. A., Perry S. J., Saeed M., Barrow M. P., O’Connor P. B. (2021). Combining
Ultraviolet Photodissociation and Two-Dimensional Mass Spectrometry:
A Contemporary Approach for Characterizing Singly Charged Agrochemicals. Anal. Chem..

[10] Rahman M., Marzullo B. P., Lam P. Y., Barrow M. P., Holman S. W., Ray A. D., O’Connor P. B. (2024). Unveiling
the Intricacy of Gapmer
Oligonucleotides through Advanced Tandem Mass Spectrometry Approaches
and Scan Accumulation for 2DMS. Analyst.

[11] Xian F., Hendrickson C. L., Blakney G. T., Beu S. C., Marshall A. G. (2010). Automated
Broadband Phase Correction of Fourier Transform Ion Cyclotron Resonance
Mass Spectra. Anal. Chem..

[12] Qi Y., Thompson C. J., Van Orden S. L., O’Connor P. B. (2011). Phase Correction
of Fourier Transform Ion Cyclotron Resonance Mass Spectra Using MatLab. J. Am. Soc. Mass Spectrom..

[13] Qi Y., Barrow M. P., Li H., Meier J. E., Van Orden S. L., Thompson C. J., O’Connor P. B. (2012). Absorption-Mode:
The Next Generation
of Fourier Transform Mass Spectra. Anal. Chem..

[14] Qi Y., Barrow M. P., Van Orden S. L., Thompson C. J., Li H., Perez-Hurtado P., O’Connor P. B. (2011). Variation of the Fourier Transform
Mass Spectra Phase Function with Experimental Parameters. Anal. Chem..

[15] Kilgour D. P. A., Neal M. J., Soulby A. J., O’Connor P. B. (2013). Improved
Optimization of the Fourier Transform Ion Cyclotron Resonance Mass
Spectrometry Phase Correction Function Using a Genetic Algorithm. Rapid Commun. Mass Spectrom..

[16] Kilgour D. P. A., Wills R., Qi Y., O’Connor P. B. (2013). Autophaser:
An Algorithm for Automated Generation of Absorption Mode Spectra for
FT-ICR MS. Anal. Chem..

[17] van
Agthoven M. A., Kilgour D. P. A., Lynch A. M., Barrow M. P., Morgan T. E., Wootton C. A., Chiron L., Delsuc M.-A., O’Connor P. B. (2019). Phase Relationships in Two-Dimensional Mass Spectrometry. J. Am. Soc. Mass Spectrom..

[18] Delsuc M.-A., Breuker K., van Agthoven M. A. (2021). Phase Correction
for Absorption Mode
Two-Dimensional Mass Spectrometry. Molecules.

[19] Palasser M., Heel S. V., Delsuc M.-A., Breuker K., van Agthoven M. A. (2023). Ultra-Accurate
Correlation between Precursor and Fragment Ions in Two-Dimensional
Mass Spectrometry: Acetylated vs Trimethylated Histone Peptides. J. Am. Soc. Mass Spectrom..

[20] Chiron, L. ; Coutouly, M.-A. ; Starck, J.-P. ; Rolando, C. ; Delsuc, M.-A. SPIKE a Processing Software Dedicated to Fourier Spectroscopies. arXiv:1608.06777. arXiv.org e-Print archive https://ui.adsabs.harvard.edu/abs/2016arXiv160806777C/abstract. 2016, pp 1–13.

[21] Halper M., Delsuc M.-A., Breuker K., van Agthoven M. A. (2020). Narrowband
Modulation Two-Dimensional Mass Spectrometry and Label-Free Relative
Quantification of Histone Peptides. Anal. Chem..

[22] Polák M., Palasser M., Kádek A., Kavan D., Wootton C. A., Delsuc M.-A., Breuker K., Novák P., van Agthoven M. A. (2023). Top-Down Proteoform Analysis by 2D MS with Quadrupolar
Detection. Anal. Chem..

[23] Hambly D. M., Gross M. L. (2005). Laser Flash Photolysis
of Hydrogen Peroxide to Oxidize
Protein Solvent-Accessible Residues on the Microsecond Timescale. J. Am. Soc. Mass Spectrom..

[24] Niu, B. ; Gross, M. L. MS-Based Hydroxyl Radical Footprinting: Methodology and Application of Fast Photochemical Oxidation of Proteins (FPOP). In Mass Spectrometry-Based Chemical Proteomics, 2019.

[25] Yassaghi G., Kukačka Z., Fiala J., Kavan D., Halada P., Volný M., Novák P. (2022). Top-Down Detection of Oxidative Protein
Footprinting by Collision-Induced Dissociation, Electron-Transfer
Dissociation, and Electron-Capture Dissociation. Anal. Chem..

[26] Polák M., Yassaghi G., Kavan D., Filandr F., Fiala J., Kukačka Z., Halada P., Loginov D. S., Novák P. (2022). Utilization
of Fast Photochemical Oxidation of Proteins and Both Bottom-up and
Top-down Mass Spectrometry for Structural Characterization of a Transcription
Factor–dsDNA Complex. Anal. Chem..

[27] Polák M., Černý J., Novák P. (2024). Isotopic Depletion Increases the
Spatial Resolution of FPOP Top-Down Mass Spectrometry Analysis. Anal. Chem..

[28] Flieger M., Wurst M., Stuchlík J., Reháček Z. (1981). Isolation
and Separation of New Natural Lactam Alkaloids of Ergot by High-Performance
Liquid Chromatography. J. Chromatogr. A.

[29] Tsybin Y. O., Quinn J. P., Tsybin O. Y., Hendrickson C. L., Marshall A. G. (2008). Electron Capture Dissociation Implementation Progress
in Fourier Transform Ion Cyclotron Resonance Mass Spectrometry. J. Am. Soc. Mass Spectrom..

[30] Huffstutler C. D., Sanchez D. M., Weigand M. R., Hu H., Li X., Chegwidden A. J., Nagornov K. O., Kozhinov A. N., Tsybin Y. O., Laskin J. (2023). Multiple Selected Ion Monitoring
Mode for Sensitive
Imaging of Eicosanoids in Tissues Using Nanospray Desorption Electrospray
Ionization (Nano-DESI) Mass Spectrometry. Int.
J. Mass Spectrom..

[31] Chiron L., van Agthoven M. A., Kieffer B., Rolando C., Delsuc M.-A. (2014). Efficient
Denoising Algorithms for Large Experimental Datasets and Their Applications
in Fourier Transform Ion Cyclotron Resonance Mass Spectrometry. Proc. Natl. Acad. Sci. U.S.A..

[32] van
Agthoven M. A., Chiron L., Coutouly M.-A., Delsuc M.-A., Rolando C. (2012). Two-Dimensional ECD FT-ICR Mass Spectrometry of Peptides
and Glycopeptides. Anal. Chem..

[33] Kilgour D.
P. A., Van Orden S. L. (2015). Absorption
Mode Fourier Transform Mass Spectrometry
with No Baseline Correction Using a Novel Asymmetric Apodization Function. Rapid Commun. Mass Spectrom..

[34] Bray F., Bouclon J., Chiron L., Witt M., Delsuc M.-A., Rolando C. (2017). Nonuniform Sampling
Acquisition of Two-Dimensional
Fourier Transform Ion Cyclotron Resonance Mass Spectrometry for Increased
Mass Resolution of Tandem Mass Spectrometry Precursor Ions. Anal. Chem..

[35] Ledford E. B., Rempel D. L., Gross M. L. (1984). Space Charge
Effects in Fourier Transform
Mass Spectrometry. II. Mass Calibration. Anal.
Chem..

[36] Palasser, M. https://github.com/michael-palasser/FAST-MS.

[37] Palasser M., Breuker K. (2025). FAST MS: Software for
the Automated Analysis of Top-Down
Mass Spectra of Polymeric Molecules Including RNA, DNA, and Proteins. J. Am. Soc. Mass Spectrom..

[38] Zubarev, R. A. Electron Capture Dissociation of Large Biomolecules. In Dissociative Recombination, Theory, Experiments and Applications IV, Proceedings of the Conference 2000; pp 214–217.

[39] Floris F., van Agthoven M., Chiron L., Soulby A. J., Wootton C. A., Lam Y. P. Y., Barrow M. P., Delsuc M.-A., O’Connor P. B. (2016). 2D FT-ICR
MS of Calmodulin: A Top-Down and Bottom-Up Approach. J. Am. Soc. Mass Spectrom..

[40] Delsuc M.-A. (1988). Spectral
Representation of 2D NMR Spectra by Hypercomplex Numbers. J. Magn. Reson..

[41] van
Agthoven M. A., Coutouly M.-A., Rolando C., Delsuc M.-A. (2011). Two-Dimensional
Fourier Transform Ion Cyclotron Resonance Mass Spectrometry: Reduction
of Scintillation Noise Using Cadzow Data Processing. Rapid Commun. Mass Spectrom..

[42] Wootton C. A., Lam Y. P. Y., Willetts M., van Agthoven M. A., Barrow M. P., Sadler P. J., O’Connor P. B. (2017). Automatic
Assignment of Metal-Containing Peptides in Proteomic LC-MS and MS/MS
Data Sets. Analyst.

[43] Flieger M., Wurst M., Shelby R. (1997). Ergot Alkaloids
— Sources,
Structures and Analytical Methods. Folia Microbiol..

[44] Scott P. M. (2007). Analysis
of Ergot Alkaloids — a Review. Mycotoxin
Res..

[45] Lehner A. F., Craig M., Fannin N., Bush L., Tobin T. (2004). Fragmentation
Patterns of Selected Ergot Alkaloids by Electrospray Ionization Tandem
Quadrupole Mass Spectrometry. J. Mass Spectrom..

