# Supplementary material for: Absorption Mode Broadband 2D MS for Proteomics and Metabolomics
Source: J Am Soc Mass Spectrom. 2026 Apr 27;37(5):1207–17. doi: 10.1021/jasms.6c00007 (PMC13235632; doi:10.1021/jasms.6c00007)
Supplement: Supplementary file 1 [file js6c00007_si_001.pdf]

Absorption mode broadband 2D MS for proteomics and metabolomics

Supporting Information

Maria A. van Agthoven[1,2,3*], Marek Polák[2,4], Jan Fiala[2,4], Claude Nelcy Ounounou[1], Petr Halada[2], Michael Palasser[3], Anne Briot-Dietsch[5], Alan Kádek[2], Kathrin Breuker[3], Petr Novák[2,4], Carlos Afonso[1], Marc-André Delsuc[5,6]

[1]Université de Rouen-Normandie, Laboratoire CARMeN UMR 6064, IRCOF, 1 rue Tesnière, Mont St Aignan Cedex, France

[2]Institute of Microbiology, Czech Academy of Sciences, Prague 14220, Czech Republic

[3]Center for Chemistry and Biomedicine, University of Innsbruck, Innrain 80/82, 6020 Innsbruck, Austria

[4]Faculty of Science, Charles University in Prague, Prague 12843, Czech Republic

[5]CASC4DE, Pôle API, 300 Boulevard Sébastien Brant, 67400 Illkirch-Graffenstaden, France

[6]Institut de Génétique et de Biologie Moléculaire et Cellulaire, INSERM U596, CNRS UMR 7104, Université de Strasbourg, 1 rue Laurent Fries, 67404 Illkirch-Graffenstaden, France

* Corresponding author: maria.van-agthoven@univ-rouen.fr

Table of contents

Figure S1. 2D ECD mass spectrum of oxidized ubiquitin. p. 3

Table S1. Fragment assignments for $[M+10H]^{10+}$ in magnitude mode. p. 4

Scheme S1. Sequence coverage in magnitude mode. p. 7

Table S2. Fragment assignments for $[M+10H]^{10+}$ in absorption mode. p. 8

Scheme S2. Sequence coverage in absorption mode. p. 12

Table S3. Fragment assignments for $[M+10H+ox]^{10+}$ in absorption mode. p. 13

Table S4. Fragment assignments for $[M+10H+2ox]^{10+}$ in absorption mode. p. 14

Table S5. Fragment assignments for $[M+9H+ox]^{9+}$ in absorption mode. p. 15

Table S6. Fragment assignments for $[M+9H+2ox]^{9+}$ in absorption mode. p. 16

Table S7. Fragment assignments for $[M+8H+ox]^{8+}$ in absorption mode. p. 17

Table S8. Fragment assignments for $[M+8H+2ox]^{8+}$ in absorption mode. p. 19

Table S9. Fragment assignments for $[M+7H+ox]^{7+}$ in absorption mode. p. 20

Table S10. Fragment assignments for $[M+7H+2ox]^{7+}$ in absorption mode. p. 22

Figure S2. Mass spectrum of oxidized ubiquitin. p. 23

Figure S3. Zoom on the 10+ charge state of the mass spectrum of oxidized ubiquitin. p. 23

Figure S4. Mass spectrum of the ergot alkaloids extracts p. 24

Table S11. Peak assignments of the mass spectrum of the ergot alkaloid extract. p.24

Table S12. Fragment assignments extracted at *m/z* 576.4 in absorption mode p. 25

Table S13. Fragment assignments at *m/z* 576.4 in magnitude mode. p. 27

Table S14. Fragment assignments at *m/z* 582.3 in absorption mode p. 29

Table S15. Fragment assignments at *m/z* 582.3 in magnitude mode p. 29

Table S16. Fragment assignments extracted at *m/z* 592.3 in absorption mode p. 30

Table S17. Fragment assignments extracted at *m/z* 592.3 in magnitude mode p. 32

Table S18. Fragment assignments extracted at *m/z* 610.3 in absorption mode p. 33

Table S19. Fragment assignments extracted at *m/z* 610.3 in magnitude mode p. 35

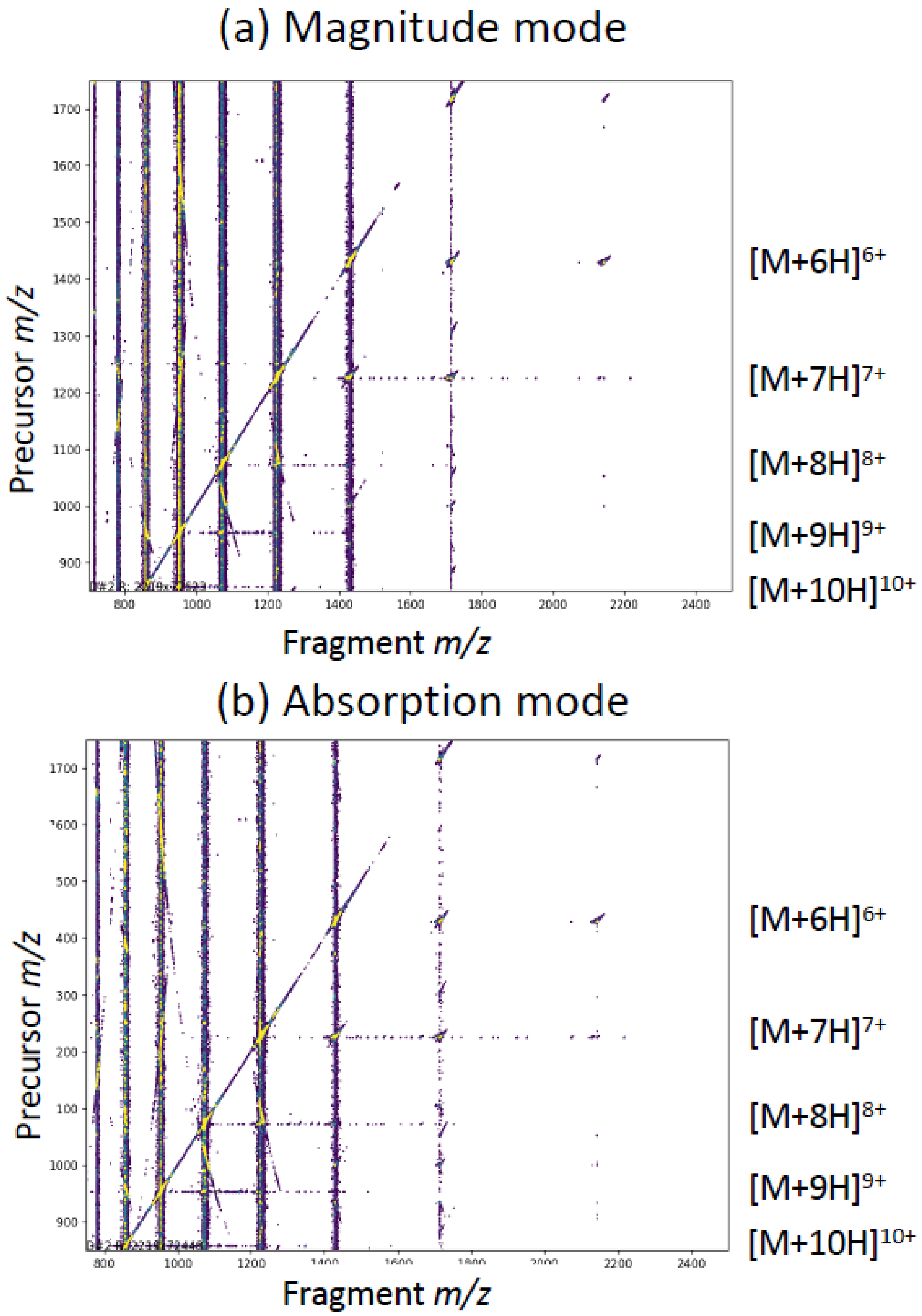

Figure S1. 2D ECD mass spectrum of oxidized ubiquitin after (a) magnitude mode processing and (b) absorption mode processing.

Table S1. Fragment assignments for $[M+10H]^{10+}$ extracted at *m/z* 857 from the magnitude mode 2D mass spectrum of oxidized ubiquitin.

m/z ratio	Assignment	Mass accuracy (ppm)	Signal-to-noise
273.14316	z_3^{+}	0.02	1.83
277.13286	c_2^{+}	-0.09	2.97
386.22768	z_4^{+}	1.20	1.38
390.21690	c_3^{+}	-0.13	2.88
640.04134	z_{17}^{3+}	-0.64	2.50
653.39860	z_{12}^{2+}	1.82	2.16
655.41266	z_6^{+}	0.41	1.49
694.39729	z_{18}^{3+}	1.60	3.82
715.88982	a_{26}^{4+}	-0.05	4.33
717.92037	z_{13}^{2+}	2.31	2.80
720.43537	$a_6^{+\bullet}$	0.36	1.52
764.44905	c_6^{+}	0.40	3.28
790.46390	c_{14}^{2+}	-2.21	2.45
822.65596	z_{36}^{5+}	0.36	5.32
828.12573	c_{44}^{6+}	0.07	10.78
833.12435	z_{22}^{3+}	-2.49	6.94
837.22240	c_{30}^{4+}	-0.72	4.31
838.46604	y_{22}^{3+}	0.05	3.92
839.46245	z_{52}^{7+}	0.37	30.37
848.26675	z_{37}^{5+}	-0.75	10.47
852.47031	$c_{45}^{6+\bullet}$	1.42	8.73
856.96867	$[M+10H]^{10+}$	-0.32	15092.26
864.47620	c_{46}^{6+}	-0.46	8.63
865.66652	$c_{39}^{5+\bullet}$	-4.61	8.65
867.33139	c_{54}^{7+}	0.28	7.37
868.57131	$z_8^{+\bullet}$	-1.59	2.34
869.23791	c_{31}^{4+}	0.30	19.98
870.30102	z_{46}^{6+}	0.16	4.86
873.97913	c_{47}^{6+}	-1.19	7.33
881.76768	c_{55}^{7+}	1.31	6.83
883.58255	y_8^{+}	-1.19	1.50
887.82435	a_{48}^{6+}	-1.58	4.84
887.99216	$a_{48}^{6+\bullet}$	-1.77	4.02
888.48819	z_{55}^{7+}	1.28	10.40
889.15280	z_{47}^{6+}	5.14	3.97
890.77453	y_{55}^{7+}	-1.02	17.52
895.33119	c_{48}^{6+}	2.08	4.48
898.15329	c_{24}^{3+}	-0.90	7.85
900.48584	z_{32}^{4+}	0.03	6.44
905.06256	$z_{56}^{7+\bullet}$	-0.97	8.61
910.20960	$c_{57}^{7+\bullet}$	-1.06	8.07

910.49596	z_{48}^{6+}	-1.57	5.99
913.11857	z_{65}^{8+}	0.43	14.75
913.16591	y_{48}^{6+}	-1.39	4.96
917.09619	c_{41}^{5+}	0.75	5.22
917.35244	z_{57}^{7+}	-0.19	6.23
922.33709	z_{49}^{6+}	0.20	11.67
926.78540	c_{58}^{7+}	-1.68	10.09
929.13075	z_{66}^{8+}	0.76	19.86
930.01727	c_{33}^{4+}	-0.92	12.32
935.51820	c_{50}^{6+}	-1.69	7.58
935.73546	z_{75}^{9+}	-0.46	5.34
935.84668	$z_{75}^{9+\bullet}$	-1.27	5.68
936.16926	c_{25}^{3+}	0.91	8.96
937.51293	y_{75}^{9+}	-3.00	8.19
939.18108	$c_{74}^{9+\bullet}$	-2.10	8.80
939.29248	c_{74}^{9+}	-2.71	8.97
940.74189	$a_{75}^{9+\bullet}$	1.64	13.40
942.51034	z_{25}^{3+}	0.79	9.39
943.68247	z_{50}^{6+}	-3.80	9.31
945.51734	$c_{75}^{9+\bullet}$	-1.51	89.02
945.62910	c_{75}^{9+}	-1.74	8.13
947.84927	y_{25}^{3+}	0.11	21.14
949.52398	a_{51}^{6+}	0.72	20.49
949.65282	z_{59}^{7+}	0.91	21.78
949.69207	$a_{51}^{6+\bullet}$	0.85	16.60
950.18420	$[M+10H-NH_3]^{9+}$	0.25	64.13
950.29570	$[M+10H-NH_3]^{9+\bullet}$	-0.26	153.09
952.07544	$[M+9H]^{9+}$	-0.38	450.77
957.02468	z_{34}^{4+}	-3.31	7.70
957.02564	c_{51}^{6+}	-1.28	25.15
959.55928	z_{17}^{2+}	0.31	12.68
960.19870	z_{51}^{6+}	1.28	8.88
961.06392	c_{17}^{2+}	0.77	6.31
962.76626	c_{68}^{8+}	0.64	24.94
963.80466	z_{60}^{7+}	0.08	6.09
963.94791	$z_{60}^{7+\bullet}$	-0.67	3.44
969.92359	z_{43}^{5+}	0.09	13.38
975.65566	z_{70}^{8+}	0.51	14.80
976.53200	c_{35}^{4+}	-2.19	5.26
977.65768	y_{70}^{8+}	0.18	21.63
979.20390	z_{52}^{6+}	-0.73	24.93
985.53354	$c_{53}^{6+\bullet}$	-0.10	6.97
985.70171	c_{53}^{6+}	0.11	6.59
989.28476	c_{70}^{8+}	0.06	32.58
991.66703	z_{71}^{8+}	0.08	19.09

993.54833	c_{44}^{5+}	-1.03	10.03
995.54151	z_{44}^{5+}	-0.99	7.81
996.05126	z_{35}^{4+}	-1.88	8.80
998.39414	z_{62}^{7+}	0.04	24.11
998.74515	y_{44}^{5+}	-1.09	11.48
1000.68333	y_{62}^{7+}	0.84	41.92
1000.70953	z_{53}^{6+}	-2.19	6.09
1003.42021	c_{71}^{8+}	0.16	6.92
1004.05058	z_{72}^{8+}	-0.03	21.31
1004.21558	a_{54}^{6+}	0.69	9.98
1004.38341	$a_{54}^{6+\bullet}$	0.56	11.98
1011.71988	c_{54}^{6+}	1.40	25.80
1012.83001	z_{63}^{7+}	0.52	7.79
1018.54816	z_{45}^{5+}	0.27	24.17
1019.12148	c_{63}^{7+}	-0.31	11.31
1021.75169	y_{45}^{5+}	0.06	13.06
1022.43413	z_{73}^{8+}	-0.37	43.51
1022.93285	c_{72}^{8+}	0.25	31.81
1022.96252	c_{45}^{5+}	-0.51	7.82
1028.06867	z_{36}^{4+}	0.87	5.04
1028.55829	y_{27}^{3+}	0.22	5.12
1035.56825	c_{28}^{3+}	-0.31	9.45
1036.56964	z_{74}^{8+}	-0.50	89.05
1037.55646	c_{64}^{7+}	-1.54	40.67
1041.09090	z_{18}^{2+}	0.25	5.57
1044.16117	z_{46}^{5+}	1.50	8.91
1048.57236	c_{47}^{5+}	-2.28	7.25
1049.98960	c_{65}^{7+}	0.36	24.94
1053.07854	$c_{38}^{4+\bullet}$	-0.99	12.25
1058.08100	a_{75}^{8+}	-2.90	17.44
1060.08021	z_{37}^{4+}	-2.08	16.64
1063.70868	c_{75}^{8+}	-3.02	119.71
1082.08357	c_{39}^{4+}	-4.35	12.92
1088.83553	z_{38}^{4+}	-3.32	10.30
1106.61125	y_{19}^{2+}	-2.01	4.67
1108.26080	c_{59}^{6+}	0.79	33.05
1114.10321	c_{40}^{4+}	0.26	12.30
1115.96484	c_{30}^{3+}	2.92	6.53
1127.60877	z_{30}^{3+}	-1.23	4.71
1130.46719	c_{70}^{7+}	0.30	17.23
1136.65219	c_{10}^{+}	2.25	7.22
1139.22650	a_{51}^{5+}	-1.79	10.61
1146.11756	c_{41}^{4+}	-1.06	15.79
1152.63445	a_{21}^{2+}	4.20	4.43
1158.64727	c_{31}^{3+}	-0.44	9.45

1164.62524	z_{62}^{6+}	0.35	11.30
1167.45839	c_{62}^{6+}	3.89	8.58
1168.49623	$z_{73}^{7+\bullet}$	-0.87	13.43
1175.13947	c_{21}^{2+}	-0.74	8.16
1184.50712	$z_{74}^{7+\bullet}$	0.68	20.50
1196.99009	c_{32}^{3+}	5.25	4.51
1200.31182	z_{32}^{3+}	-0.14	4.91
1200.64607	$z_{32}^{3+\bullet}$	-1.55	5.56
1209.23550	a_{75}^{7+}	-4.19	16.12
1215.52317	c_{75}^{7+}	-4.95	34.56
1264.74572	c_{11}^{+}	0.89	3.86
1282.20230	c_{23}^{2+}	-3.05	4.59
1329.71150	c_{59}^{5+}	0.25	10.10
1346.72497	c_{24}^{2+}	-1.88	19.48
1413.76924	$z_{25}^{2+\bullet}$	3.23	7.57

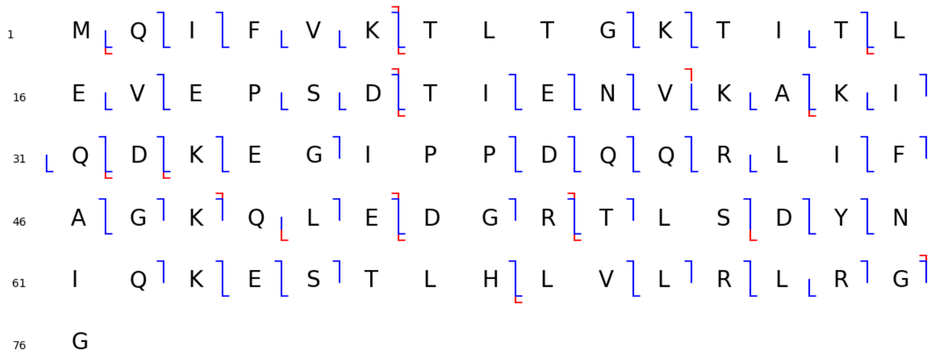

Scheme S1. Sequence coverage obtained from the fragmentation pattern in Fig. 3a in magnitude mode.

Table S2. Fragment assignments for $[M+10H]^{10+}$ extracted at *m/z* 857 from the absorption mode 2D mass spectrum of oxidized ubiquitin.

m/z ratio	Assignment	Mass accuracy (ppm)	Signal-to-noise
273.14300	z_3^{+}	-0.56	6.26
277.13280	c_2^{+}	-0.31	10.05
386.22710	z_4^{+}	-0.32	5.70
390.21697	c_3^{+}	0.04	8.29
502.81593	z_9^{2+}	0.79	3.00
537.28548	c_4^{+}	0.22	3.56
542.32839	z_5^{+}	0.10	2.16
559.35758	z_{10}^{2+}	0.03	3.14
592.34001	$a_5^{+\bullet}$	-0.22	2.19
609.88157	z_{11}^{2+}	0.28	7.27
636.35381	c_5^{+}	0.05	6.66
640.04188	z_{17}^{3+}	0.22	8.36
653.39738	z_{12}^{2+}	-0.06	7.21
655.41234	z_6^{+}	-0.08	6.18
694.39675	z_{18}^{3+}	0.81	12.28
717.91938	z_{13}^{2+}	0.93	7.22
720.43594	$a_6^{+\bullet}$	1.16	3.48
733.07426	$z_{19}^{3+\bullet}$	-0.25	3.39
764.44933	c_6^{+}	0.77	8.74
781.96606	z_{14}^{2+}	-0.17	5.16
789.97612	y_{14}^{2+}	0.71	4.03
790.46553	c_{14}^{2+}	-0.14	5.57
809.11187	$c_{43}^{6+\bullet}$	1.87	2.95
809.27897	c_{43}^{6+}	0.79	3.47
822.65642	z_{36}^{5+}	0.92	11.94
828.12582	c_{44}^{6+}	0.18	26.05
833.12688	z_{22}^{3+}	0.55	15.65
837.22372	c_{30}^{4+}	0.85	5.35
838.46605	y_{22}^{3+}	0.06	6.42
839.46255	z_{52}^{7+}	0.48	28.39
842.86697	c_{38}^{5+}	0.34	14.97
845.99573	z_{15}^{2+}	0.29	4.75
847.00733	c_{15}^{2+}	-0.41	6.48
848.26778	z_{37}^{5+}	0.47	16.20
864.47684	c_{46}^{6+}	0.28	8.21
865.49677	c_7^{+}	0.40	2.33
867.18807	$c_{54}^{7+\bullet}$	1.03	7.79
867.56459	z_8^{+}	-0.32	5.80
869.23737	c_{31}^{4+}	-0.32	38.72
870.30050	z_{46}^{6+}	-0.44	9.25
873.81370	$c_{47}^{6+\bullet}$	1.71	7.56

873.98097	c_{47}^{6+}	0.91	6.78
881.62392	$c_{55}^{7+\bullet}$	1.55	4.55
881.76716	c_{55}^{7+}	0.71	2.54
885.16033	z_{23}^{3+}	0.23	11.65
886.99132	$a_{32}^{4+\bullet}$	0.39	7.95
887.82605	a_{48}^{6+}	0.33	6.04
887.99315	$a_{48}^{6+\bullet}$	-0.65	5.01
888.48736	z_{55}^{7+}	0.35	13.81
890.77573	y_{55}^{7+}	0.33	30.37
895.16380	$c_{48}^{6+\bullet}$	2.72	4.08
895.33090	c_{48}^{6+}	1.75	3.09
898.15456	c_{24}^{3+}	0.51	15.03
900.48611	z_{32}^{4+}	0.32	11.71
904.91947	z_{56}^{7+}	0.00	7.50
910.21272	$c_{57}^{7+\bullet}$	2.36	6.91
910.49836	z_{48}^{6+}	1.07	12.73
911.52994	c_{16}^{2+}	1.07	4.77
913.11836	z_{65}^{8+}	0.20	10.10
916.89552	$c_{41}^{5+\bullet}$	1.72	5.49
917.09639	c_{41}^{5+}	0.96	6.11
922.33680	z_{49}^{6+}	-0.11	20.57
926.64343	$c_{58}^{7+\bullet}$	0.48	10.57
926.78666	c_{58}^{7+}	-0.32	6.17
929.13129	z_{66}^{8+}	1.33	25.06
930.01877	c_{33}^{4+}	0.69	25.52
932.71117	z_{41}^{5+}	0.51	8.65
935.35285	$c_{50}^{6+\bullet}$	1.12	4.53
935.51996	c_{50}^{6+}	0.20	3.34
936.16919	c_{25}^{3+}	0.84	16.25
940.62964	a_{75}^{9+}	1.36	10.37
940.74105	$a_{75}^{9+\bullet}$	0.75	7.03
942.51053	z_{25}^{3+}	0.98	27.42
943.68599	z_{50}^{6+}	-0.07	8.27
943.85339	$z_{50}^{6+\bullet}$	-0.68	5.83
944.11529	z_{42}^{5+}	0.32	6.04
945.51856	$c_{75}^{9+\bullet}$	-0.22	106.55
949.52468	a_{51}^{6+}	1.46	32.99
949.65276	z_{59}^{7+}	0.85	55.77
949.69190	$a_{51}^{6+\bullet}$	0.67	24.08
949.79609	$z_{59}^{7+\bullet}$	0.17	12.20
950.07459	$[M+10H-H_2O]^{9+}$	-0.04	15.70
950.18534	$[M+10H-H_2O]^{9+\bullet}$	-1.34	158.71
950.29600	$[M+10H-NH3]^{9+\bullet}$	0.07	51.54
952.07539	$[M+10H]^{9+}$	-0.43	418.22
956.86016	$c_{51}^{6+\bullet}$	1.31	25.42

957.02712	z_{34}^{4+}	-0.76	7.29
957.02748	c_{51}^{6+}	0.64	19.27
959.55930	z_{17}^{2+}	0.34	52.05
960.19887	z_{51}^{6+}	1.46	28.07
961.06206	c_{17}^{2+}	-1.17	21.50
962.64083	$c_{68}^{8+\bullet}$	1.21	22.81
962.76636	c_{68}^{8+}	0.74	17.98
963.80498	z_{60}^{7+}	0.41	9.51
963.94823	$z_{60}^{7+\bullet}$	-0.34	4.35
969.19192	c_{26}^{3+}	0.73	7.29
969.92407	z_{43}^{5+}	0.58	30.40
975.65543	z_{70}^{8+}	0.28	10.57
976.19891	c_{52}^{6+}	0.90	6.25
976.28289	$c_{35}^{4+\bullet}$	0.72	4.86
977.65760	y_{70}^{8+}	0.10	28.53
979.20545	z_{52}^{6+}	0.84	49.04
982.24113	z_{61}^{7+}	1.92	11.28
985.53461	$c_{53}^{6+\bullet}$	0.98	12.58
985.70170	c_{53}^{6+}	0.10	8.40
989.15928	$c_{70}^{8+\bullet}$	0.56	23.17
989.28472	c_{70}^{8+}	0.02	18.55
991.66703	z_{71}^{8+}	0.91	24.63
993.34942	$c_{44}^{5+\bullet}$	1.64	11.81
993.54971	c_{44}^{5+}	0.36	11.28
993.54979	a_{36}^{4+}	0.00	11.82
995.54342	z_{44}^{5+}	0.93	11.42
996.05369	z_{35}^{4+}	0.56	17.51
998.39512	z_{62}^{7+}	1.02	31.90
998.74714	y_{44}^{5+}	0.90	15.40
1000.68269	y_{62}^{7+}	0.19	42.59
1000.71238	z_{53}^{6+}	0.66	12.98
1004.21556	a_{54}^{6+}	0.67	36.60
1011.55237	$c_{54}^{6+\bullet}$	1.86	13.38
1011.71917	c_{54}^{6+}	0.70	19.36
1012.82917	z_{63}^{7+}	-0.32	6.00
1018.54834	z_{45}^{5+}	0.45	52.88
1019.12114	c_{63}^{7+}	-0.65	8.79
1020.89748	$a_{28}^{3+\bullet}$	0.11	7.92
1021.75251	y_{45}^{5+}	0.86	23.73
1022.56011	z_{73}^{8+}	-0.95	55.44
1028.06930	z_{36}^{4+}	1.49	9.97
1028.56043	c_{55}^{6+}	0.68	9.04
1035.56640	c_{28}^{3+}	-2.10	15.08
1036.40074	z_{55}^{6+}	0.38	10.59
1036.69562	z_{74}^{8+}	-0.85	109.26

1037.55646	c_{64}^{7+}	-0.07	52.26
1039.77082	$a_{47}^{5+\bullet}$	-1.16	6.47
1041.09143	z_{18}^{2+}	0.75	15.92
1044.15980	z_{46}^{5+}	0.19	20.18
1048.37405	$c_{47}^{5+\bullet}$	0.82	4.23
1048.57457	c_{47}^{5+}	-0.17	3.69
1050.13358	c_{65}^{7+}	-0.37	36.48
1052.57696	z_{75}^{8+}	0.57	34.41
1058.08100	a_{75}^{8+}	-2.15	18.73
1060.08232	z_{37}^{4+}	-0.09	27.51
1063.70868	c_{75}^{8+}	-1.17	132.02
1066.77556	z_{47}^{5+}	-0.80	5.78
1066.97611	$z_{47}^{5+\bullet}$	-1.75	3.97
1071.24516	y_{28}^{3+}	0.85	7.42
1078.26596	c_{29}^{3+}	-0.87	7.57
1079.62640	c_{9}^{+}	-1.64	4.64
1081.08358	c_{58}^{6+}	2.47	41.46
1081.83863	$c_{39}^{4+\bullet}$	2.14	7.86
1082.08917	c_{39}^{4+}	0.82	12.50
1088.83813	z_{38}^{4+}	-0.94	16.99
1092.39541	z_{48}^{5+}	0.71	18.96
1106.61214	y_{19}^{2+}	-1.21	10.50
1108.09283	c_{59}^{6+}	2.04	64.02
1113.94186	y_{29}^{3+}	-0.64	3.74
1114.10325	c_{40}^{4+}	0.29	23.02
1115.96261	c_{30}^{3+}	0.92	15.90
1117.62488	c_{20}^{2+}	-1.78	12.04
1127.61151	z_{30}^{3+}	1.21	9.65
1130.46719	c_{70}^{7+}	-0.23	18.46
1132.42339	z_{50}^{5+}	1.56	6.19
1133.18985	z_{71}^{7+}	-0.47	7.71
1136.65051	c_{10}^{+}	0.78	23.09
1143.97743	$a_{31}^{3+\bullet}$	0.76	4.80
1146.11756	c_{41}^{4+}	0.01	27.40
1148.23079	c_{51}^{5+}	0.04	13.10
1151.28971	z_{31}^{3+}	0.46	3.19
1151.62395	$z_{31}^{3+\bullet}$	-1.02	2.59
1153.13446	$a_{21}^{2+\bullet}$	0.81	13.41
1158.64691	c_{31}^{3+}	-0.75	20.81
1159.95482	a_{62}^{6+}	2.81	8.69
1164.62524	z_{62}^{6+}	1.58	15.50
1174.63870	$c_{21}^{2+\bullet}$	1.93	9.02
1175.14217	c_{21}^{2+}	1.55	12.05
1184.50712	z_{74}^{7+}	0.01	26.79
1196.98947	c_{32}^{3+}	-0.52	7.36

1198.66231	z_{21}^{2+}	0.13	6.91
1200.31197	z_{32}^{3+}	-0.02	19.22
1200.64956	$z_{32}^{3+\bullet}$	1.36	5.80
1209.09153	a_{75}^{7+}	-0.30	26.13
1215.52317	c_{75}^{7+}	-0.01	39.89
1249.18691	z_{22}^{2+}	0.73	10.99
1257.19500	y_{22}^{2+}	-0.28	4.13
1264.74565	c_{11}^{+}	0.84	7.34
1272.93304	z_{45}^{4+}	1.41	16.55
1282.20601	c_{23}^{2+}	-0.16	12.12
1346.72807	c_{24}^{2+}	0.41	52.71
1403.74953	c_{25}^{2+}	0.40	3.96
1413.76488	$z_{25}^{2+\bullet}$	0.15	4.73
1478.87844	c_{13}^{+}	1.43	2.81
1579.92405	c_{14}^{+}	0.02	7.59
1920.11757	$c_{17}^{+\bullet}$	3.29	4.41
1921.12182	c_{17}^{+}	1.42	2.05

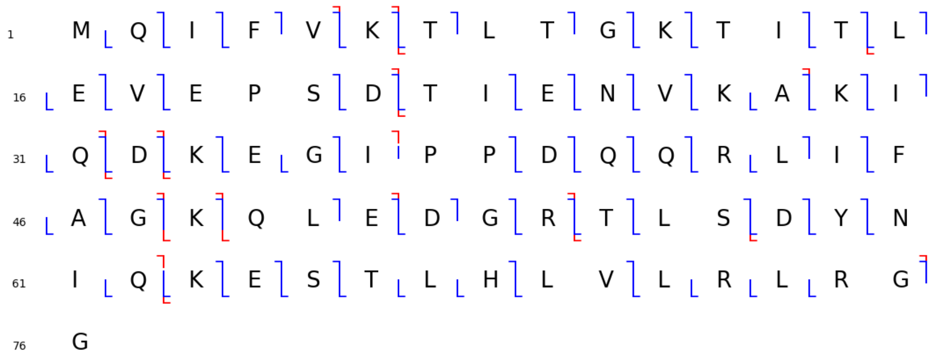

Scheme S2. Sequence coverage obtained from the fragmentation pattern in Fig. 3a in absorption mode.

Table S3. Fragment assignments for $[M+10H+ox]^{10+}$ extracted at *m/z* 859 from the absorption mode 2D mass spectrum of oxidized ubiquitin.

***m/z* ratio**	**Assignment**	**Mass accuracy (ppm)**	**Signal-to-noise**
273.14308	z_3^{+}	-0.28	2.30
386.22726	z_4^{+}	0.10	3.00
609.88136	z_{11}^{2+}	-0.07	4.95
640.04172	z_{17}^{3+}	-0.03	4.51
694.39662	z_{18}^{3+}	0.62	4.33
764.44904	c_6^{+}	0.38	3.29
768.45922	$a_{14}^{2+\bullet}$	0.51	5.78
833.12756	z_{22}^{3+}	1.36	6.44
841.60316	$(z_{52}+ox)^{7+}$	-0.03	10.34
868.98648	$c_{31}^{4+\bullet}$	0.90	11.10
900.23386	z_{32}^{4+}	1.77	6.85
931.12886	$(z_{66}+ox)^{8+}$	-0.59	10.34
950.08417	c_{59}^{7+}	2.57	6.98
950.29692	$[M+10H-NH_3]^{9+\bullet}$	1.03	28.99
951.96340	$[M+10H+ox-NH_3]^{9+}$	2.34	113.69
953.85190	$[M+10H+ox]^{9+}$	-1.16	131.07
961.06347	c_{17}^{2+}	0.30	14.66
977.65518	$(z_{70}+ox)^{8+}$	0.67	9.43
979.20582	z_{52}^{6+}	1.23	12.78
981.86837	$(z_{52}+ox)^{6+}$	-2.11	15.06
991.15940	$(c_{70}+ox)^{8+\bullet}$	1.32	7.74
1000.68143	y_{62}^{7+}	-1.07	18.50
1000.71207	z_{53}^{6+}	0.35	8.19
1002.96722	$(y_{62}+ox)^{7+}$	-0.26	14.79
1004.21670	a_{54}^{6+}	1.82	5.62
1004.38357	$a_{54}^{6+\bullet}$	0.71	3.71
1011.88816	c_{27}^{3+}	-1.36	12.65
1020.89582	$a_{28}^{3+\bullet}$	-1.51	4.27
1021.74888	$(z_{45}+ox)^{5+}$	1.97	11.45
1024.43350	$(z_{73}+ox)^{8+}$	-0.04	17.77
1035.56823	c_{28}^{3+}	-0.34	2.05
1038.56901	$(z_{74}+ox)^{8+}$	-3.16	24.80
1065.70804	$(c_{75}+ox)^{7+}$	-1.72	76.23
1148.90705	$(c_{71}+ox)^{7+}$	0.20	14.43
1200.31036	z_{32}^{3+}	-1.36	8.51
1346.72658	c_{24}^{2+}	-0.69	16.99
1354.22106	$(c_{24}+ox)^{2+\bullet}$	1.24	4.45
1413.10472	$(a_{75}+ox)^{6+}$	-2.26	13.92

Table S4. Fragment assignments for $[M+10H+2ox]^{10+}$ extracted at *m/z* 861 from the absorption mode 2D mass spectrum of oxidized ubiquitin.

m/z ratio	Assignment	Mass accuracy (ppm)	Signal-to-noise
273.14296	z_3^{+}	-0.73	1.81
390.21690	c_3^{+}	-0.14	1.92
559.35738	z_{10}^{2+}	-0.34	1.78
636.35399	c_5^{+}	0.33	1.76
798.03871	$(a_{36}+ox)^{5+}$	1.02	6.06
868.98687	$c_{31}^{4+\bullet}$	1.36	8.27
942.51011	z_{25}^{3+}	0.55	6.44
953.73842	$[M+9H+2ox-NH_3]^{9+}$	0.03	53.70
953.84982	$[M+10H+2ox-NH_3]^{9+\bullet}$	-0.57	46.21
955.62991	$[M+9H+2ox]^{9+}$	-0.33	128.45
955.74131	$[M+9H+2ox]^{9+\bullet}$	-0.94	57.69
999.94852	$(c_{44}+2ox)^{5+}$	1.20	6.30
1002.96616	$(y_{62}+ox)^{7+}$	-1.32	8.34
1026.43286	$(z_{73}+2ox)^{8+}$	0.65	15.42
1040.56837	$(z_{74}+2ox)^{8+}$	-0.47	19.42
1042.12643	$(c_{64}+2ox)^{7+}$	1.11	9.62

Table S5. Fragment assignments for $[M+9H+ox]^{9+}$ extracted at *m/z* 954 from the absorption mode 2D mass spectrum of oxidized ubiquitin.

m/z ratio	Assignment	Mass accuracy (ppm)	Signal-to-noise
273.14305	z_3^+	-0.37	3.87
277.13281	c_2^+	-0.28	4.60
386.22728	z_4^+	0.16	1.61
558.32355	$(z_5+ox)^+$	0.54	1.18
981.86992	$(z_{52}+ox)^{6+}$	-0.53	26.17
1041.73650	$(y_{55}+ox)^{6+}$	0.52	5.57
1045.70506	$(z_{65}+ox)^{7+}$	0.32	6.97
1047.35938	$(z_{46}+ox)^{5+}$	0.76	10.92
1053.33236	c_{38}^{4+}	0.78	11.02
1054.57474	$(z_{75}+ox)^{8+}$	-1.51	19.15
1068.95523	$[M+9H-NH_3]^{8+\bullet}$	-1.68	34.03
1072.95756	$[M+9H+ox]^{8+}$	-1.09	171.03
1091.58697	$(y_{58}+ox)^{6+}$	-0.86	5.93
1092.83832	$(z_{38}+ox)^{4+}$	0.40	7.75
1095.59574	$(z_{48}+ox)^{5+}$	1.23	14.56
1102.30432	$(c_{68}+ox)^{7+\bullet}$	3.28	13.24
1110.42563	$(z_{59}+ox)^{6+}$	0.37	19.56
1110.92695	$(c_{59}+ox)^{6+}$	0.30	13.98
1119.46395	$(y_{70}+ox)^{7+}$	0.00	13.76
1132.61133	$(c_{70}+ox)^{7+\bullet}$	2.76	22.20
1136.64974	c_{10}^+	0.09	11.48
1142.42601	$(a_{51}+ox)^{5+}$	0.46	9.13
1148.44755	$(z_{61}+ox)^{6+}$	0.77	7.20
1151.22725	$(c_{51}+ox)^{5+\bullet}$	-0.83	14.37
1158.31359	$c_{31}^{3+\bullet}$	1.52	6.55
1170.12421	$(c_{62}+ox)^{6+}$	2.50	29.69
1170.63724	$(z_{73}+ox)^{7+}$	-0.25	25.90
1186.79211	$(z_{74}+ox)^{7+}$	-1.09	26.71
1212.98047	$(c_{64}+ox)^{6+}$	-1.01	17.81
1217.80815	$(c_{75}+ox)7^+$	-3.92	80.75
1282.70202	c_{34}^{3+}	-0.46	4.81
1332.91049	$(c_{59}+ox)^{5+}$	2.22	14.03
1354.22342	$(c_{24}+ox)^{2+\bullet}$	1.75	6.80
1354.72503	$(c_{24}+ox)^{2+}$	0.05	3.77
1365.57557	$(z_{73}+ox)^{6+}$	-1.45	10.48
1384.42292	$(z_{74}+ox)^{6+}$	2.44	10.74
1921.12513	c_{17}^+	3.15	4.10

Table S6. Fragment assignments for $[M+9H+2ox]^{9+}$ extracted at *m/z* 956 from the absorption mode 2D mass spectrum of oxidized ubiquitin.

***m/z* ratio**	**Assignment**	**Mass accuracy (ppm)**	**Signal-to-noise**
273.14307	z_3^+	-0.30	2.14
636.35367	c_5^+	-0.17	1.81
720.43520	$a_6^{+\bullet}$	-1.37	10.49
739.94074	C_{13}^{2+}	-1.44	2.0
984.53431	$(z_{52}+2ox)^{6+}$	-1.98	10.59
1041.73298	$(z_{55}+2ox)^{6+}$	0.96	7.21
1056.57596	$(z_{75}+2ox)^{8+}$	0.25	12.50
1056.69862	$(z_{75}+2ox)^{8+\bullet}$	-2.89	2.44
1064.08044	$(z_{37}+ox)4^+$	-1.53	2.06
1066.28652	$(z_{66}+2ox)7^+$	-2.32	6.08
1069.33090	$[M+9H-NH_3]^{8+\bullet\bullet\bullet\bullet}$	-3.79	122.74
1072.83031	$[M+9H+2ox-NH_3]^{8+}$	0.50	19.51
1072.95753	$[M+9H+2ox-NH_3]^{8+\bullet}$	-2.82	17.56
1074.95753	$[M+9H+2ox]^{8+}$	-0.52	156.00
1086.24651	$(c_{58}+2ox)^{6+\bullet}$	-0.68	5.51
1086.41489	$(c_{58}+2ox)^{6+}$	-0.30	7.74
1104.58910	$(c_{68}+2ox)^{7+\bullet}$	3.08	3.64
1104.73233	$(c_{68}+2ox)^{7+}$	2.40	6.50
1113.09121	$(z_{59}+2ox)^{6+}$	0.16	8.49
1113.42597	$(c_{59}+2ox)^{6+\bullet}$	1.05	12.85
1113.59274	$(c_{59}+2ox)^{6+}$	0.28	6.10
1119.46008	$(z_{70}+2ox)^{7+}$	-0.42	9.24
1121.74906	$(y_{70}+2ox)^{7+}$	0.11	9.21
1134.89417	$(c_{70}+2ox)^{7+\bullet}$	0.86	4.95
1135.03740	$(c_{70}+2ox)^{7+}$	0.20	7.88
1154.42884	$(c_{51}+2ox)^{5+\bullet}$	1.43	4.94
1154.62929	$(c_{51}+2ox)^{5+}$	0.46	5.22
1169.95688	$(z_{62}+2ox)^{6+}$	1.51	12.29
1172.79003	$(c_{62}+2ox)^{6+}$	2.42	10.17
1172.92223	$(z_{73}+2ox)^{7+}$	-1.20	17.97
1189.07710	$(z_{74}+2ox)^{7+}$	0.61	19.30
1189.64706	$(c_{73}+2ox)^{7+}$	1.56	10.41

Table S7. Fragment assignments for [M+8H+ox]$^{8+}$ extracted at *m/z* 1073 from the absorption mode 2D mass spectrum of oxidized ubiquitin.

***m/z* ratio**	**Assignment**	**Mass accuracy (ppm)**	**Signal-to-noise**
273.14298	z_3^+	-0.64	2.13
277.13280	c_2^+	-0.31	3.20
386.22714	z_4^+	-0.20	2.88
390.21694	c_3^+	-0.03	3.76
537.28541	c_4^+	0.07	2.22
636.35348	c_5^+	-0.47	1.65
655.41195	z_6^+	-0.68	2.05
720.43481	$a_6^{\bullet+}$	-0.40	3.12
764.44867	c_6^+	-0.10	4.59
959.55955	z_{17}^{2+}	0.59	15.32
960.06369	$z_{17}^{2+\bullet}$	0.84	19.25
1041.09182	z_{18}^{2+}	1.13	8.16
1057.07948	$(c_{38}+ox)^{4+\bullet}$	1.11	14.16
1060.08016	z_{37}^{4+}	-2.12	18.73
1069.08112	$[M+8H-NH_3]^{8+\bullet\bullet}$	-1.76	69.41
1088.83838	z_{38}^{4+}	-0.70	14.39
1092.39482	z_{48}^{5+}	-0.54	8.46
1092.83786	$(z_{38}+ox)^{4+}$	-0.02	10.44
1095.59651	$(z_{48}+ox)^{5+}$	1.93	15.02
1136.65111	c_{10}^+	1.31	8.64
1139.22485	a_{51}^{5+}	-1.45	5.19
1142.42448	$(a_{51}+ox)^{5+}$	-0.88	8.28
1167.29393	y_{62}^{6+}	-0.94	10.91
1169.95948	$(y_{62}+ox)^{6+}$	-1.17	4.70
1170.12699	$(c_{62}+ox)^{6+}$	2.37	5.94
1174.84335	z_{52}^{5+}	-0.63	10.24
1175.14186	c_{21}^{2+}	1.29	7.58
1178.04368	$(z_{52}+ox)^{5+}$	0.51	8.18
1179.89233	z_{42}^{4+}	0.35	6.89
1184.14156	$(z_{42}+ox)^{4+\bullet}$	-0.88	5.98
1196.99120	c_{32}^{3+}	0.93	7.22
1200.64488	$z_{32}^{3+\bullet}$	-2.55	6.11
1205.08747	$(z_{75}+ox)^{7+}$	1.06	20.66
1211.37291	$(a_{75}+ox)^{7+}$	-2.98	8.85
1211.51552	$(a_{75}+ox)^{7+\bullet}$	-4.10	5.98
1215.37997	$c_{75}^{7+\bullet}$	0.64	23.06
1217.66419	$(c_{75}+ox)^{7+\bullet}$	0.01	116.92
1221.52038	$[M+8H-NH_3]^{7+\bullet}$	-0.74	17.47
1223.66596	$[M+8H+ox-H_2O]^{7+\bullet}$	0.22	98.38
1223.80904	$[M+7H]^{7+}$	-0.51	212.14
1226.23752	$[M+8H+ox]^{7+\bullet}$	-0.90	94.76

1241.17129	$(z_{66}\text{+ox})^{6+}$	0.94	14.60
1244.17658	z_{44}^{4+}	0.22	10.37
1248.17540	$(z_{44}\text{+ox})^{4+}$	0.30	8.18
1248.42610	$(z_{44}\text{+ox})^{4+\bullet}$	-0.71	2.86
1249.18526	z_{22}^{2+}	-0.59	6.95
1264.74495	c_{11}^{+}	0.28	5.87
1272.93339	z_{45}^{4+}	0.28	4.02
1273.18408	$z_{45}^{4+\bullet}$	-0.72	6.23
1276.93113	$(z_{45}\text{+ox})^{4+}$	-0.50	7.67
1286.01652	$(c_{68}\text{+ox})^{6+}$	-0.83	8.40
1303.20361	$(z_{70}\text{+ox})^{6+}$	1.39	18.15
1305.87339	$(y_{70}\text{+ox})^{6+}$	-0.38	14.99
1321.37633	$(c_{70}\text{+ox})^{6+}$	0.43	11.02
1329.51058	$c_{59}^{5+\bullet}$	0.48	17.33
1332.71053	$(c_{59}\text{+ox})^{5+\bullet}$	1.21	24.74
1346.72789	c_{24}^{2+}	0.28	13.32
1365.57557	$(z_{73}\text{+ox})^{6+}$	0.02	14.47
1366.07256	$(c_{72}\text{+ox})^{6+}$	0.75	14.27
1384.42292	$(z_{74}\text{+ox})^{6+}$	0.87	20.94
1385.08787	$(c_{73}\text{+ox})^{6+}$	0.26	22.34
1389.43508	a_{38}^{3+}	1.42	6.92
1405.76601	$(z_{75}\text{+ox})^{6+}$	-0.34	10.64
1413.10313	z_{37}^{3+}	-3.06	7.09
1413.26776	z_{25}^{2+}	4.95	11.44
1413.43738	$z_{37}^{3+\bullet}$	-4.26	3.73
1417.94248	c_{75}^{6+}	-2.16	19.32
1420.60830	$(c_{75}\text{+ox})^{6+}$	-2.14	66.38
1442.11016	$c_{39}^{3+\bullet}$	-1.74	8.17
1442.44438	c_{39}^{3+}	-2.93	5.72
1447.77851	$(c_{39}\text{+ox})^{3+}$	-1.20	2.73
1539.82066	c_{68}^{5+}	2.49	7.60
1543.63490	$(z_{69}\text{+ox})^{5+\bullet}$	-1.56	9.06
1552.84743	c_{28}^{2+}	-1.15	9.84
1638.69080	$(z_{73}\text{+ox})^{5+}$	0.11	9.07
1661.13546	z_{59}^{4+}	1.80	11.88
1665.88629	$(c_{59}\text{+ox})^{4+}$	-0.63	12.21
1704.52851	$(c_{75}\text{+ox})^{5+}$	-2.32	13.20
1736.96335	$c_{31}^{2+\bullet}$	-0.44	4.01
1737.46467	c_{31}^{2+}	-1.93	2.88
1921.11857	c_{17}^{+}	-0.26	6.70

Table S8. Fragment assignments for $[M+8H+2ox]^{8+}$ extracted at *m/z* 1076 from the absorption mode 2D mass spectrum of oxidized ubiquitin.

m/z ratio	Assignment	Mass accuracy (ppm)	Signal-to-noise
277.13268	c_2^{+}	-0.74	1.59
386.22713	z_4^{+}	-0.22	1.19
390.21675	c_3^{+}	-0.51	1.91
764.44886	c_6^{+}	0.15	1.47
1041.09091	z_{18}^{2+}	0.26	3.30
1219.94794	$(c_{75}+2ox)^{7+\bullet}$	-1.01	65.69
1221.94730	$[M+4H-NH_3]^{7+\bullet\bullet\bullet\bullet}$	-4.83	88.16
1225.80661	$[M+7H+2ox-H_2O]^{7+}$	-0.08	54.02
1226.09273	$[M+7H+2ox-NH_3]^{7+\bullet}$	1.20	103.34
1228.37899	$[M+7H+2ox]^{7+}$	-0.53	303.51
1243.83911	$(y_{66}+ox)^{6+}$	-0.65	10.79
1305.70146	$(z_{70}+2ox)^{6+}$	2.20	11.64
1308.53921	$(y_{70}+2ox)^{6+}$	-0.88	10.10
1324.04215	$(c_{70}+2ox)^{6+}$	-0.26	8.90
1332.50850	$(z_{59}+ox)^{5+\bullet}$	-1.40	6.34
1335.70920	$(z_{59}+2ox)^{5+\bullet}$	-0.11	5.75
1335.90979	$(c_{59}+2ox)^{5+\bullet}$	1.41	9.63
1346.22431	$c_{24}^{2+\bullet}$	0.53	9.41
1346.72562	c_{24}^{2+}	-1.41	2.96
1365.57557	$(z_{73}+ox)^{6+}$	0.12	7.79
1368.40936	$(z_{73}+2ox)^{6+}$	-2.35	9.98
1387.08874	$(z_{74}+2ox)^{6+}$	0.49	11.40
1423.27412	$(c_{75}+2ox)^{6+}$	-1.16	39.88

Table S9. Fragment assignments for $[M+7H+ox]^{7+}$ extracted at *m/z* 1226 from the absorption mode 2D mass spectrum of oxidized ubiquitin.

m/z ratio	Assignment	Mass accuracy (ppm)	Signal-to-noise
277.13278	c_2^+	-0.41	3.14
636.35373	c_5^+	-0.09	1.65
764.44878	c_6^+	0.05	5.93
1041.09128	z_{18}^{2+}	0.61	4.12
1079.62957	c_9^+	1.30	2.97
1221.23427	$[M+7H-H_2O]^{7+}$	-2.01	63.09
1346.72802	c_{24}^{2+}	0.38	5.46
1400.55019	y_{62}^{5+}	-1.71	6.05
1405.76592	$(z_{75}+ox)^{6+}$	-0.06	13.12
1410.60248	$a_{75}^{6+\bullet}$	-3.12	8.85
1410.93669	$(c_{74}+ox)^{6+\bullet}$	-0.04	5.81
1413.26349	z_{25}^{2+}	1.93	5.62
1413.26889	$(a_{75}+ox)^{6+\bullet}$	-2.69	9.49
1417.76860	$c_{75}^{6+\bullet}$	-4.17	11.38
1425.44186	$[M+3H-NH_3]^{6+\bullet\bullet\bullet\bullet}$	-1.63	33.48
1427.43935	$[M+6H+ox-NH_3]^{6+}$	0.86	91.75
1430.27590	$[M+6H+ox]^{6+}$	0.01	412.83
1489.19937	$(z_{66}+ox)^{5+}$	-2.24	21.55
1500.60650	$(z_{67}+ox)^{5+}$	-0.32	9.89
1542.82347	$(c_{68}+ox)^{5+\bullet}$	3.49	8.94
1543.02400	$(c_{68}+ox)^{5+}$	2.82	8.15
1563.64287	$(z_{70}+ox)^{5+}$	0.10	18.82
1589.06030	$(z_{71}+ox)^{5+}$	-0.47	10.09
1638.48923	$(z_{73}+ox)^{5+}$	-3.56	10.07
1639.28718	$(c_{72}+ox)^{5+}$	1.12	12.99
1661.10604	$(z_{74}+ox)^{5+}$	-0.80	32.39
1665.38614	$(z_{59}+ox)^{4+}$	-0.71	35.08
1686.71776	$(z_{75}+ox)^{5+}$	-0.30	12.87
1695.52421	$(a_{75}+ox)^{5+}$	-0.22	12.43
1829.22781	$(z_{65}+ox)^{4+}$	0.04	11.12
1875.50692	$(z_{67}+ox)^{4+}$	-0.12	8.50
1928.77470	$(c_{68}+ox)^{4+}$	1.47	13.12
1954.55373	$(z_{70}+ox)^{4+}$	1.20	14.41
1982.07483	z_{71}^{4+}	-2.23	10.17
2047.85972	$(z_{73}+ox)^{4+}$	0.49	8.4
2076.13074	$(z_{74}+ox)^{4+}$	-0.84	14.67
2077.12817	$(c_{73}+ox)^{4+}$	0.02	9.38
2130.15686	$(c_{75}+ox)^{4+}$	-2.40	29.29
2219.84316	$(z_{59}+ox)^{3+}$	-0.38	13.64

Table S10. Fragment assignments for $[M+7H+2ox]^{7+}$ extracted at *m/z* 1229 from the absorption mode 2D mass spectrum of oxidized ubiquitin.

***m/z* ratio**	**Assignment**	**Mass accuracy (ppm)**	**Signal-to-noise**
273.14303	z_3^+	-0.48	1.16
390.21684	c_3^+	-0.28	1.28
1136.64987	c_{10}^+	0.22	3.21
1276.93316	$(z_{45}+ox)^{4+}$	1.09	4.56
1408.43300	$(z_{75}+2ox)^{6+}$	0.83	13.22
1425.43926	$[M+3H-NH_3]^{6+\bullet\bullet\bullet\bullet}$	-3.46	139.68
1429.93931	$[M+6H+2ox-H_2O]^{6+}$	-0.44	47.63
1430.10642	$[M+6H+2ox-H_2O]^{6+\bullet}$	-1.04	45.54
1432.94138	$[M+6H+2ox]^{6+}$	-0.22	394.71
1503.80537	$(z_{67}+2ox)^{5+}$	-0.40	7.59
1524.81955	$(c_{54}+2ox)^{4+\bullet}$	-1.87	6.95
1546.01788	$(c_{68}+2ox)^{5+\bullet}$	0.53	9.22
1566.84186	$(z_{70}+2ox)^{5+}$	-0.55	12.42
1588.64913	$(c_{70}+2ox)^{5+}$	1.17	7.56
1642.08303	$(c_{72}+2ox)^{5+}$	3.03	10.39
1664.30503	$(z_{74}+2ox)^{5+}$	-0.67	18.73
1665.10297	$(c_{73}+2ox)^{5+}$	-0.37	17.88
1669.63683	$(z_{59}+2ox)^{4+}$	-1.53	18.35
1704.52851	$(c_{75}+ox)^{5+}$	-0.12	11.57
1707.32436	$(c_{75}+2ox)^{5+}$	-2.71	32.95
1832.97458	$(z_{65}+2ox)^{4+}$	0.40	7.53
1865.25028	$(z_{66}+2ox)^{4+}$	-1.20	7.85
1958.30050	$(z_{70}+2ox)^{4+}$	0.50	8.74
2080.38142	$(z_{74}+2ox)^{4+}$	-2.44	9.06

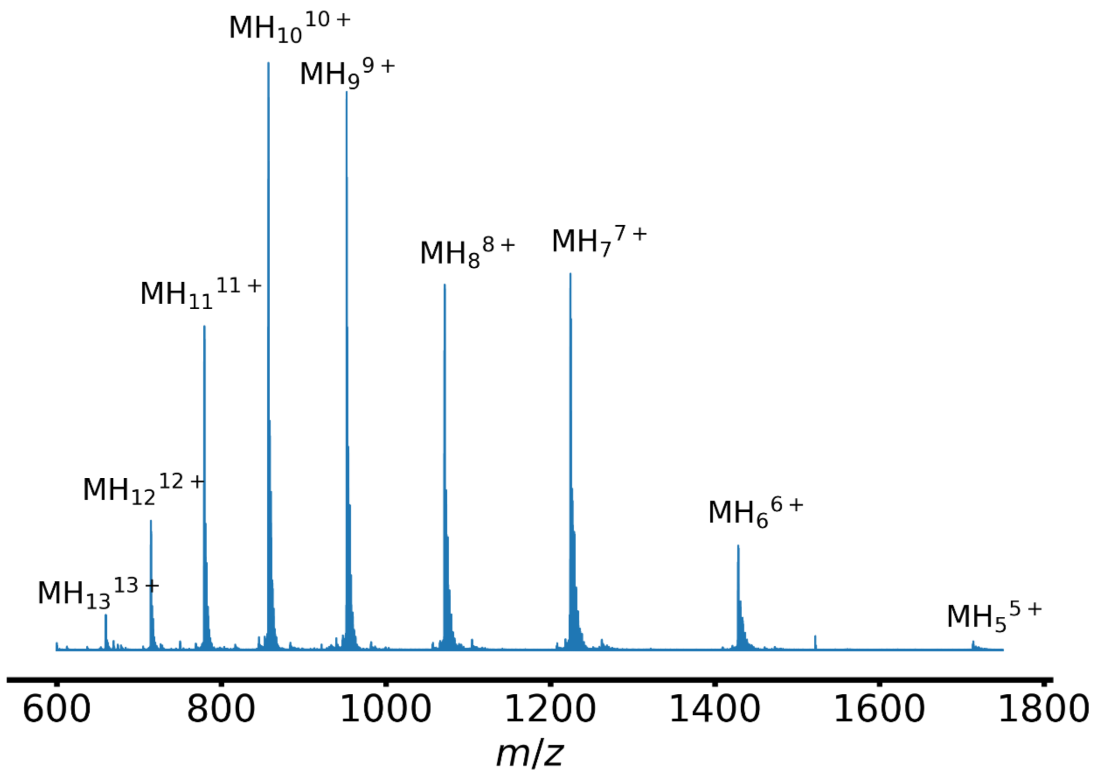

Figure S2. Mass spectrum of oxidized ubiquitin.

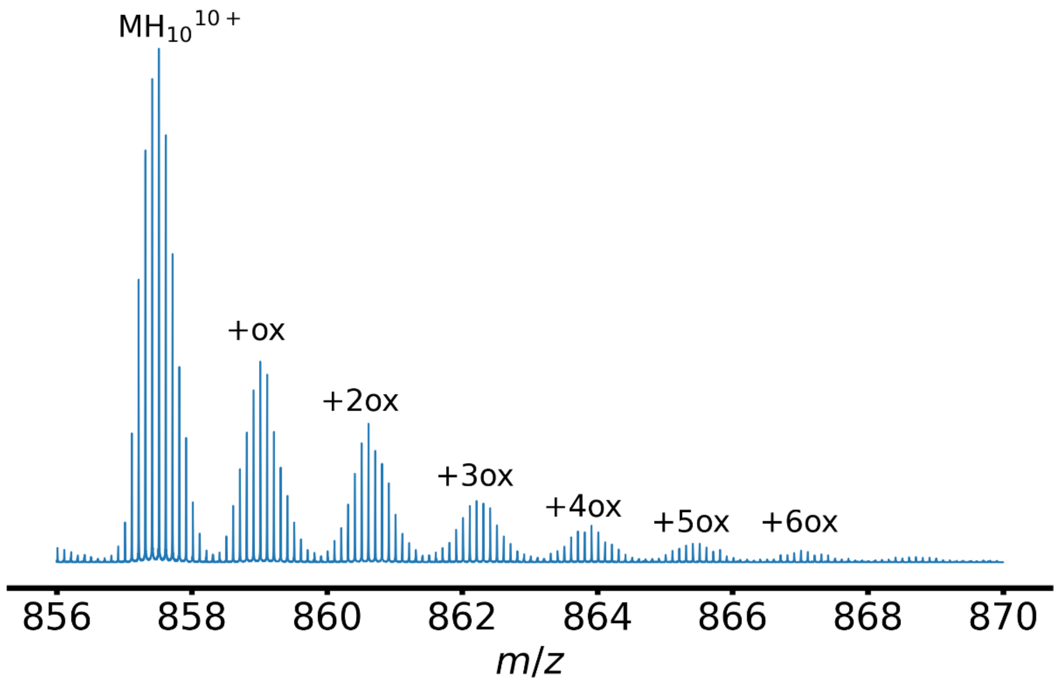

Figure S3. Zoom on the 10+ charge state of the mass spectrum of oxidized ubiquitin.

Figure S4. Phase-corrected absorption mode mass spectrum of the ergot alkaloid abstract after CASI isolation.

Table S11. Peak assignments of the mass spectrum of the ergot alkaloid extract.

Experimental m/z	Assignment	$[M+H]^+$ ion	Mass Accuracy (ppm)
534.271114	Ergovaline/ergobutine	$C_{29}H_{36}N_5O_5^+$	0.03
548.286683	Ergosine α/β	$C_{30}H_{38}N_5O_5^+$	-0.11
562.302423	Ergocornine/ergoptine α/β	$C_{31}H_{40}N_5O_5^+$	0.05
576.318037	Ergocryptine α/β	$C_{32}H_{42}N_5O_5^+$	-0.02
582.271173	Ergotamine	$C_{33}H_{36}N_5O_5^+$	0.13
592.255421	Unknown	$C_{34}H_{34}N_5O_5^+$	-0.04
592.291815	Ergocristine dehydrate	$C_{35}H_{38}N_5O_4^+$	-0.03
610.302337	Ergocristine	$C_{35}H_{40}N_5O_5^+$	-0.10

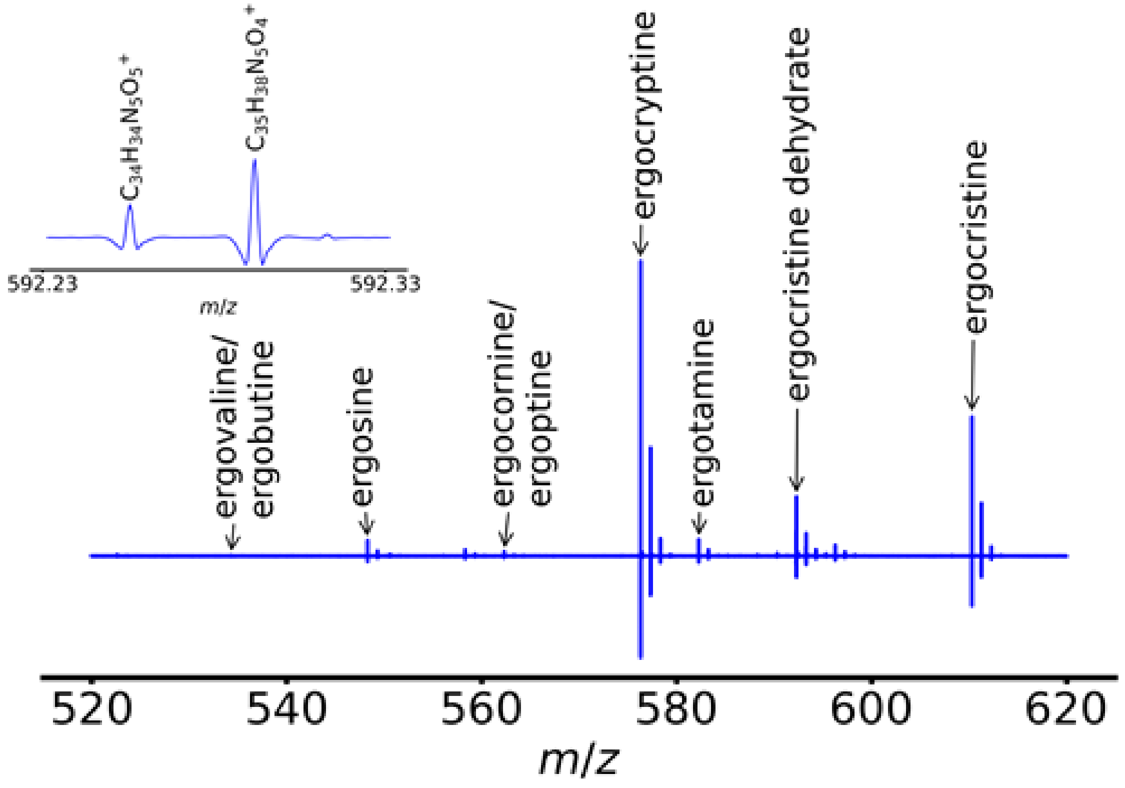

Table S12. Fragment assignments extracted at *m/z* 576.4 (ergocryptine, $C_{32}H_{42}N_5O_5^+$) from the absorption mode 2D mass spectrum of ergot alkaloid extracts.

**Precursor *m/z***	**Fragment *m/z***	**Assignment**	**Fragment mass accuracy (ppm)**
576.31631	151.05422	$C_{12}H_7^+$	-0.02
576.30584	152.06205	$C_{12}H_8^+$	0.00
576.31871	153.06984	$C_{12}H_9^+$	-0.21
576.30280	154.06511	$C_{11}H_7N^+$	-0.13
576.31996	165.06989	$C_{13}H_9^+$	0.07
576.30945	167.07296	$C_{12}H_9N^+$	0.03
576.30687	168.08079	$C_{12}H10N^+$	0.06
576.31012	178.06514	$C_{13}H_8N^+$	0.10
576.31206	179.07297	$C_{13}H_9N^+$	0.09
576.30961	180.08078	C_{13} $H10N^+$	0.02
576.29187	181.13351	$C_{10}H_{17}N_2O^+$	-0.17
576.30625	182.09643	$C_{13}H_{12}N^+$	0.01
576.30517	183.14924	$C_{10}H_{19}N_2O^+$	0.26
576.31401	190.06512	$C_{14}H_8N^+$	-0.03
576.30478	191.07294	$C_{14}H_9N^+$	-0.07
576.30918	192.08077	$C_{14}H_{10}N^+$	-0.03
576.31862	193.08860	$C_{14}H_{11}N^+$	0.00
576.30899	194.09643	$C_{14}H_{12}N^+$	0.00
576.30537	195.09167	$C_{13}H_{11}N_2^+$	-0.04
576.31148	196.09949	$C_{13}H_{12}N_2^+$	-0.05
576.31186	197.10732	$C_{13}H_{13}N_2^+$	-0.02
576.29649	205.07604	$C_{14}H_9N_2^+$	0.06
576.30000	206.06005	$C_{14}H_8NO^+$	0.05
576.30311	206.08384	$C_{14}H_{10}N_2^+$	-0.03
576.31143	207.06787	$C_{14}H_9NO^+$	0.01
576.30607	207.09168	$C_{14}H_{11}N_2^+$	0.03
576.30635	208.07568	$C_{14}H_{10}NO^+$	-0.07
576.31071	208.09950	$C_{14}H_{12}N_2^+$	0.01
576.30395	209.07094	$C_{13}H_9N_2O^+$	0.00
576.30497	209.12842	$C_{11}H_{17}N_2O_2^+$	-0.15
576.30568	211.1441	$C_{11}H_{19}N_2O_2^+$	-0.12
576.30931	220.07569	$C_{15}H_{10}NO^+$	0.02
576.30658	220.09952	$C_{15}H_{12}N_2^+$	0.11
576.30721	221.10733	$C_{15}H_{13}N_2^+$	0.03
576.31071	222.11515	$C_{15}H_{14}N_2^+$	-0.01
576.30809	223.12296	$C_{15}H_{15}N_2^+$	-0.05
576.31205	224.09443	$C_{14}H_{12}N_2O^+$	0.09
576.30747	224.13090	$C_{15}H_{16}N_2^+$	0.43
576.30803	225.10223	$C_{14}H_{13}N_2O^+$	-0.03
576.31595	237.10221	$C_{15}H_{13}N_2O^+$	-0.12

576.28642	249.10214	$C_{16}H_{13}N_2O^+$	-0.39
576.30834	251.11793	$C_{16}H_{15}N_2O^+$	0.14
576.31345	259.12303	$C_{18}H_{15}N_2^+$	0.21
576.30603	261.13861	$C_{18}H_{17}N_2^+$	-0.06
576.31450	263.17550	$C_{15}H_{23}N_2O_2^+$	0.37
576.30215	266.12886	$C_{16}H_{16}N_3O^+$	0.28
576.30854	267.13661	$C_{16}H_{17}N_3O^+$	-0.01
576.30807	268.14446	$C_{16}H_{18}N_3O^+$	0.09
576.31012	277.13360	$C_{18}H_{17}N_2O^+$	0.23
576.30956	281.18599	$C_{15}H_{25}N_2O_3^+$	0.07
576.31922	287.11795	$C_{19}H_{15}N_2O^+$	0.22
576.30834	291.17034	$C_{16}H_{23}N_2O_3^+$	0.08
576.31404	304.12058	$C_{19}H_{16}N_2O_2^+$	-0.13
576.30848	305.12844	$C_{19}H_{17}N_2O_2^+$	-0.07
576.31159	307.14426	$C_{19}H_{19}N_2O_2^+$	0.49
576.31529	319.16794	$C_{20}H_{21}N_3O^+$	0.07
576.30879	320.17569	$C_{20}H_{22}N_3O^+$	-0.16
576.33186	338.18629	$C_{20}H_{24}N_3O_2^+$	-0.03
576.30935	347.16280	$C_{21}H_{21}N_3O_2^+$	-0.09
576.30796	348.17065	$C_{21}H_{22}N_3O_2^+$	0.00
576.30494	454.19993	$C_{27}H_{26}N_4O_3^+$	-0.03
576.30808	557.29928	$C_{32}H_{39}N_5O_4^+$	-0.68

Average of absolute value of mass accuracy (fragment *m/z*): 0.10 ppm

Precursor *m/z* range: 45 mDa (average: *m/z* 576.30873)

Precursor *m/z* standard deviation: 6 mDa

Note : the precision of the precursor m/z should be 1 decimal place. The excessive number of decimals was chosen to highlight how far the reader should trust the data.

Table S13. Fragment assignments at *m/z* 576.4 (ergocryptine, $C_{32}H_{42}N_5O_5^+$) from the magnitude mode 2D mass spectrum of ergot alkaloid extracts.

Precursor *m/z*	Fragment *m/z*	Assignment	Fragment mass accuracy (ppm)
576.22342	151.05424	$C_{12}H_7^+$	0.11
576.33235	152.06209	$C_{12}H_8^+$	0.22
576.33979	153.06991	$C_{12}H_9^+$	0.19
576.38493	154.06524	$C_{11}H_7N^+$	0.71
576.34545	165.06997	$C_{13}H_9^+$	0.57
576.28366	167.07286	$C_{12}H_9N^+$	-0.54
576.27740	168.08086	$C_{12}H_{10}N^+$	0.52
576.29393	178.06518	$C_{13}H_8N^+$	0.30
576.27824	179.07300	$C_{13}H_9N^+$	0.25
576.31983	180.08082	$C_{13}H_{10}N^+$	0.26
576.25427	181.13355	$C_{10}H_{17}N_2O^+$	0.04
576.39243	182.09641	$C_{13}H_{12}N^+$	-0.06
576.25409	183.14927	$C_{10}H_{19}N_2O^+$	0.46
576.30833	190.06521	$C_{14}H_8N^+$	0.46
576.32040	191.07298	$C_{14}H_9N^+$	0.14
576.30279	192.08084	$C_{14}H_{10}N^+$	0.32
576.31449	193.08865	$C_{14}H_{11}N^+$	0.27
576.31194	194.09648	$C_{14}H_{12}N^+$	0.30
576.38580	195.09176	$C_{13}H_{11}N_2^+$	0.42
576.31429	196.09955	$C_{13}H_{12}N_2^+$	0.24
576.29545	197.10740	$C_{13}H_{13}N_2^+$	0.36
576.28105	205.07606	$C_{14}H_9N_2^+$	0.15
576.32701	206.06008	$C_{14}H_8NO^+$	0.21
576.27725	206.08400	$C_{14}H_{10}N_2^+$	0.74
576.31118	207.06791	$C_{14}H_9NO^+$	0.20
576.30388	207.09172	$C_{14}H_{11}N_2^+$	0.23
576.30488	208.07575	$C_{14}H_{10}NO^+$	0.27
576.32065	208.09953	$C_{14}H_{12}N_2^+$	0.15
576.35767	209.07101	$C_{13}H_9N_2O^+$	0.35
576.30199	209.12843	$C_{11}H_{17}N_2O_2^+$	-0.12
576.30187	211.14417	$C_{11}H_{19}N_2O_2^+$	0.29
576.36685	220.07545	$C_{15}H_{10}NO^+$	-1.08
576.34986	220.09957	$C_{15}H_{12}N_2^+$	0.33
576.31405	221.10733	$C_{15}H_{13}N_2^+$	0.03
576.30503	222.11517	$C_{15}H_{14}N_2^+$	0.11
576.30561	223.12303	$C_{15}H_{15}N_2^+$	0.23
576.32425	224.09442	$C_{14}H_{12}N_2O^+$	0.01
576.35010	224.13095	$C_{15}H_{16}N_2^+$	0.66
576.30173	225.10230	$C_{14}H_{13}N_2O^+$	0.28
576.31502	237.10216	$C_{15}H_{13}N_2O^+$	-0.32

576.23574	249.10267	$C_{16}H_{13}N_2O^+$	1.75
576.30638	251.11793	$C_{16}H_{15}N_2O^+$	0.15
576.31500	261.13867	$C_{18}H_{17}N_2^+$	0.17
576.31309	263.17551	$C_{15}H_{23}N_2O_2^+$	0.40
576.38256	266.12878	$C_{16}H_{16}N_3O^+$	-0.04
576.31531	267.13660	$C_{16}H_{17}N_3O^+$	-0.07
576.31014	268.14445	$C_{16}H_{18}N_3O^+$	0.04
576.32512	277.13355	$C_{18}H_{17}N_2O^+$	0.05
576.34103	281.18571	$C_{15}H_{25}N_2O_3^+$	-0.91
576.33878	287.11785	$C_{19}H_{15}N_2O^+$	-0.14
576.31181	291.17038	$C_{16}H_{23}N_2O_3^+$	0.22
576.32706	304.12071	$C_{19}H_{16}N_2O_2^+$	0.27
576.30750	305.12853	$C_{19}H_{17}N_2O_2^+$	0.23
576.40574	307.14410	$C_{19}H_{19}N_2O_2^+$	-0.01
576.32401	319.16809	$C_{20}H_{21}N_3O^+$	0.54
576.29327	320.17536	$C_{20}H_{22}N_3O^+$	-1.19
576.44317	338.18662	$C_{20}H_{24}N_3O_2^+$	0.93
576.31133	347.16275	$C_{21}H_{21}N_3O_2^+$	-0.24
576.30795	348.17071	$C_{21}H_{22}N_3O_2^+$	0.16
576.31363	454.19928	$C_{27}H_{26}N_4O_3^+$	-1.45
576.30627	557.29946	$C_{32}H_{39}N_5O_4^+$	-0.35

Average of absolute value of mass accuracy (fragment *m/z*): 0.36 ppm

Precursor *m/z* range: 220 mDa (average: *m/z* 576.31784)

Precursor *m/z* standard deviation: 38 mDa

Note : the precision of the precursor m/z should be 1 decimal place. The excessive number of decimals was chosen to highlight how far the reader should trust the data.

Table S14. Fragment assignments at *m/z* 582.3 (ergotamine, $C_{33}H_{36}N_5O_5^+$) from the absorption mode 2D mass spectrum of ergot alkaloid extracts.

**Precursor *m/z***	**Fragment *m/z***	**Assignment**	**Fragment mass accuracy (ppm)**
582.22197	305.12845	$C_{19}H_{17}N_2O_2^+$	-0.02

Note : the precision of the precursor m/z should be 1 decimal place. The excessive number of decimals was chosen to highlight how far the reader should trust the data.

Table S15. Fragment assignments at *m/z* 582.3 (ergotamine, $C_{33}H_{36}N_5O_5^+$) from the magnitude mode 2D mass spectrum of ergot alkaloid extracts.

**Precursor *m/z***	**Fragment *m/z***	**Assignment**	**Fragment mass accuracy (ppm)**
582.292088	305.128370	$C_{19}H_{17}N_2O_2^+$	-0.28

Note : the precision of the precursor m/z should be 1 decimal place. The excessive number of decimals was chosen to highlight how far the reader should trust the data.

Table S16. Fragment assignments extracted at *m/z* 592.3 (ergocristine dehydrate, $C_{35}H_{38}N_5O_4^+$, unknown compound, $C_{34}H_{34}N_5O_5^+$) from the absorption mode 2D mass spectrum of ergot alkaloid extracts.

Precursor *m/z*	Precursor assignment	Fragment *m/z*	Fragment assignment	Fragment mass accuracy (ppm)
592.25895	$C_{34}H_{34}N_5O_5^+$	151.05422	$C_{12}H_7^+$	-0.02
592.26210	$C_{34}H_{34}N_5O_5^+$	178.06515	$C_{13}H_8N^+$	0.15
592.27592	$C_{35}H_{38}N_5O_4^+$	180.08077	$C_{13}H_{10}N^+$	-0.01
592.28039	$C_{35}H_{38}N_5O_4^+$	190.06509	$C_{14}H_8N^+$	-0.17
592.29067	$C_{35}H_{38}N_5O_4^+$	191.07292	$C_{14}H_9N^+$	-0.15
592.28581	$C_{35}H_{38}N_5O_4^+$	192.08075	$C_{14}H_{10}N^+$	-0.11
592.25070	$C_{34}H_{34}N_5O_5^+$	205.07602	$C_{14}H_9N_2^+$	-0.02
592.24250	$C_{34}H_{34}N_5O_5^+$	206.08384	$C_{14}H_{10}N_2^+$	-0.05
592.28559	$C_{35}H_{38}N_5O_4^+$	207.06787	$C_{14}H_9NO^+$	0.00
592.24539	$C_{34}H_{34}N_5O_5^+$	207.09166	$C_{14}H_{11}N_2^+$	-0.08
592.28436	$C_{35}H_{38}N_5O_4^+$	221.10733	$C_{15}H_{13}N_2^+$	0.01
592.28249	$C_{35}H_{38}N_5O_4^+$	223.12297	$C_{15}H_{15}N_2^+$	-0.03
592.25209	$C_{34}H_{34}N_5O_5^+$	233.07098	$C_{15}H_9N_2O^+$	0.17
592.24411	$C_{34}H_{34}N_5O_5^+$	234.07874	$C_{15}H_{10}N_2O^+$	-0.10
592.28357	$C_{35}H_{38}N_5O_4^+$	243.11279	$C_{14}H_{15}N_2O_2^+$	-0.07
592.23887	$C_{34}H_{34}N_5O_5^+$	250.09748	$C_{15}H_{12}N_3O^+$	-0.04
592.23493	$C_{34}H_{34}N_5O_5^+$	251.08146	$C_{15}H_{11}N_2O_2^+$	-0.18
592.28324	$C_{35}H_{38}N_5O_4^+$	261.13864	$C_{18}H_{17}N_2^+$	0.05
592.29734	$C_{35}H_{38}N_5O_4^+$	268.14442	$C_{16}H_{18}N_3O^+$	-0.08
592.28871	$C_{35}H_{38}N_5O_4^+$	305.12845	$C_{19}H_{17}N_2O_2^+$	-0.02
592.28756	$C_{35}H_{38}N_5O_4^+$	325.15472	$C_{19}H_{21}N_2O_3^+$	0.16
592.24213	$C_{34}H_{34}N_5O_5^+$	330.12376	$C_{20}H_{16}N_3O_2^+$	0.18
592.28461	$C_{35}H_{38}N_5O_4^+$	348.17070	$C_{21}H_{22}N_3O_2^+$	0.14

Average of absolute value of mass accuracy (fragment *m/z*): 0.09 ppm.

Precursor *m/z* range: 62 mDa.

Precursor *m/z* standard deviation: 21 mDa.

For fragment ion peaks assigned to $C_{34}H_{34}N_5O_5^+$:

Precursor *m/z* range: 27 mDa (average precursor m/z 592.2472).

Precursor *m/z* standard deviation: 9 mDa.

For fragment ion peaks assigned to $C_{35}H_{38}N_5O_4^+$:

Precursor *m/z* range: 21 mDa (average precursor *m/z* 592.2854).

Precursor *m/z* standard deviation: 5 mDa.

Note : the precision of the precursor m/z should be 1 decimal place. The excessive number of decimals was chosen to highlight how far the reader should trust the data.

Table S17. Fragment assignments extracted at *m/z* 592.3 (ergocristine dehydrate, $C_{35}H_{38}N_5O_4^+$, unknown compound, $C_{34}H_{34}N_5O_5^+$) from the magnitude mode 2D mass spectrum of ergot alkaloid extracts.

**Precursor *m/z***	**Fragment *m/z***	**Fragment assignment**	**Fragment mass accuracy (ppm)**
592.20539	151.05428	$C_{12}H_7^+$	0.35
592.2854	178.06509	$C_{13}H_8N^+$	-0.21
592.2731	180.08086	$C_{13}H_{10}N^+$	0.45
592.25853	190.06517	$C_{14}H_8N^+$	0.26
592.30394	191.07297	$C_{14}H_9N^+$	0.12
592.27422	192.08083	$C_{14}H_{10}N^+$	0.29
592.26905	205.07605	$C_{14}H_9N_2^+$	0.12
592.24784	206.08390	$C_{14}H_{10}N_2^+$	0.25
592.28585	207.06795	$C_{14}H_9NO^+$	0.40
592.26635	207.09167	$C_{14}H_{11}N_2^+$	0.00
592.29796	221.10735	$C_{15}H_{13}N_2^+$	0.13
592.28650	223.12307	$C_{15}H_{15}N_2^+$	0.43
592.25942	233.07107	$C_{15}H_9N_2O^+$	0.56
592.30481	234.07870	$C_{15}H_{10}N_2O^+$	-0.27
592.25534	243.11280	$C_{14}H_{15}N_2O_2^+$	0.00
592.24297	250.09756	$C_{15}H_{12}N_3O^+$	0.28
592.31692	251.08183	$C_{15}H_{11}N_2O_2^+$	1.30
592.27707	261.13874	$C_{18}H_{17}N_2^+$	0.44
592.35821	268.14443	$C_{16}H_{18}N_3O^+$	-0.04
592.28299	305.12852	$C_{19}H_{17}N_2O_2^+$	0.22
592.29258	325.15470	$C_{19}H_{21}N_2O_3^+$	0.10
592.24826	330.12377	$C_{20}H_{16}N_3O_2^+$	0.21
592.32118	348.17080	$C_{21}H_{22}N_3O_2^+$	0.42

Average of absolute value of mass accuracy (fragment *m/z*): 0.30 ppm.

Precursor *m/z* range: 153 mDa (average precursor *m/z* 592.2789).

Precursor *m/z* standard deviation: 31 mDa.

Note : the precision of the precursor m/z should be 1 decimal place. The excessive number of decimals was chosen to highlight how far the reader should trust the data.

Table S18. Fragment assignments extracted at *m/z* 610.3 (ergocristine, $C_{35}H_{40}N_5O_5^+$) from the absorption mode 2D mass spectrum of ergot alkaloid extracts.

**Precursor *m/z***	**Fragment *m/z***	**Assignment**	**Fragment mass accuracy (ppm)**
610.29063	120.08075	$C_8H_{10}N^+$	-0.21
610.29668	151.05421	$C_{12}H_7^+$	-0.12
610.29000	152.06204	$C_{12}H_8^+$	-0.09
610.2927	153.06987	$C_{12}H_9^+$	-0.06
610.29072	154.06512	$C_{11}H_8N^+$	-0.06
610.29409	165.06989	$C_{13}H_9^+$	0.08
610.28800	167.07294	$C_{12}H_9N^+$	-0.05
610.28323	168.08075	$C_{12}H_{10}N^+$	-0.13
610.28916	178.06511	$C_{13}H_8N^+$	-0.10
610.28666	179.07295	$C_{13}H_9N^+$	0.00
610.28934	180.08078	$C_{13}H_{10}N^+$	0.02
610.29231	182.09643	$C_{13}H_{12}N^+$	0.04
610.28735	190.06512	$C_{14}H_8N^+$	-0.02
610.28907	191.07293	$C_{14}H_9N^+$	-0.09
610.28886	192.08077	$C_{14}H_{10}N^+$	-0.03
610.29271	193.08859	$C_{14}H_{11}N^+$	-0.06
610.29212	194.09642	$C_{14}H_{12}N^+$	-0.02
610.28888	195.09169	$C_{13}H_{11}N_2^+$	0.09
610.28883	196.09947	$C_{13}H_{12}N_2^+$	-0.14
610.29074	197.10732	$C_{13}H_{13}N_2^+$	-0.03
610.29013	205.07605	$C_{14}H_9N_2^+$	0.12
610.28806	206.06003	$C_{14}H_8NO^+$	-0.04
610.29093	206.08380	$C_{14}H_{10}N_2^+$	-0.23
610.28895	207.06786	$C_{14}H_9NO^+$	-0.03
610.28669	207.09167	$C_{14}H_{11}N_2^+$	-0.05
610.28941	208.07568	$C_{14}H_{10}NO^+$	-0.07
610.28797	208.09950	$C_{14}H_{12}N_2^+$	-0.01
610.28527	209.07094	$C_{13}H_9N_2O^+$	0.01
610.29084	217.13355	$C_{13}H_{17}N_2O^+$	0.05
610.29091	220.09948	$C_{15}H_{12}N_2^+$	-0.08
610.28902	221.10732	$C_{15}H_{13}N_2^+$	-0.03
610.28908	222.11513	$C_{15}H_{14}N_2^+$	-0.07
610.29011	223.12295	$C_{15}H_{15}N_2^+$	-0.09
610.28987	224.09442	$C_{14}H_{12}N_2O^+$	0.00
610.28904	225.10224	$C_{14}H_{13}N_2O^+$	-0.02
610.28234	237.10223	$C_{15}H_{13}N_2O^+$	-0.06
610.28788	243.11283	$C_{14}H_{15}N_2O_2^+$	0.12
610.29114	245.12845	$C_{14}H_{17}N_2O_2^+$	-0.01
610.28762	251.11790	$C_{16}H_{15}N_2O^+$	0.05
610.29036	261.13867	$C_{18}H_{17}N_2^+$	0.16

610.28943	267.13665	$C_{16}H_{17}N_3O^+$	0.14
610.28852	268.14445	$C_{16}H_{18}N_3O^+$	0.04
610.29031	277.13353	$C_{18}H_{17}N_2O^+$	-0.03
610.28709	304.12060	$C_{19}H_{16}N_2O_2^+$	-0.11
610.28857	305.12844	$C_{19}H_{17}N_2O_2^+$	-0.06
610.28555	307.14417	$C_{19}H_{19}N_2O_2^+$	0.22
610.29178	315.17028	$C_{18}H_{23}N_2O_3^+$	-0.12
610.28621	319.16792	$C_{20}H_{21}N_3O^+$	0.00
610.28262	320.17574	$C_{20}H_{22}N_3O^+$	0.00
610.28935	325.15470	$C_{19}H_{21}N_2O_3^+$	0.08
610.28872	338.18613	$C_{20}H_{24}N_3O_2^+$	-0.52
610.28189	342.18126	$C_{19}H_{24}N_3O_3^+$	0.11
610.27282	346.15487	$C_{21}H_{20}N_3O_2^+$	-0.38
610.28915	347.16285	$C_{21}H_{21}N_3O_2^+$	0.07
610.28833	348.17064	$C_{21}H_{22}N_3O_2^+$	-0.03
610.29011	488.18371	$C_{30}H_{24}N_4O_3^+$	-1.20

Average of absolute value of mass accuracy (fragment *m/z*): 0.10 ppm.

Precursor *m/z* range: 24 mDa (average precursor *m/z* 610.2887).

Precursor *m/z* standard deviation: 3 mDa.

Note : the precision of the precursor m/z should be 1 decimal place. The excessive number of decimals was chosen to highlight how far the reader should trust the data.

Table S19. Fragment assignments extracted at *m/z* 610.3 (ergocristine, $C_{35}H_{40}N_5O_5^+$) from the magnitude mode 2D mass spectrum of ergot alkaloid extracts.

Precursor *m/z*	Fragment *m/z*	Assignment	Fragment mass accuracy (ppm)
610.34006	120.08082	$C_8H_{10}N^+$	0.37
610.31046	151.05430	$C_{12}H_7^+$	0.47
610.32627	152.06212	$C_{12}H_8^+$	0.43
610.28989	153.06994	$C_{12}H_9^+$	0.40
610.29773	154.06518	$C_{11}H_8N^+$	0.37
610.25772	165.06991	$C_{13}H_9^+$	0.20
610.26839	167.07302	$C_{12}H_9N^+$	0.39
610.32465	168.08084	$C_{12}H_{10}N^+$	0.39
610.29011	178.06517	$C_{13}H_8N^+$	0.25
610.27242	179.07302	$C_{13}H_9N^+$	0.37
610.28807	180.08083	$C_{13}H_{10}N^+$	0.31
610.29032	182.09657	$C_{13}H_{12}N^+$	0.81
610.29481	190.06517	$C_{14}H_8N^+$	0.24
610.30334	191.07300	$C_{14}H_9N^+$	0.25
610.29555	192.08084	$C_{14}H_{10}N^+$	0.32
610.31222	193.08864	$C_{14}H_{11}N^+$	0.19
610.29639	194.09649	$C_{14}H_{12}N^+$	0.31
610.22875	195.09183	$C_{13}H_{11}N_2^+$	0.81
610.29013	196.09953	$C_{13}H_{12}N_2^+$	0.16
610.27296	197.10734	$C_{13}H_{13}N_2^+$	0.08
610.29147	205.07616	$C_{14}H_9N_2^+$	0.65
610.29295	206.05995	$C_{14}H_8NO^+$	-0.45
610.24299	206.08397	$C_{14}H_{10}N2^+$	0.57
610.29274	207.06792	$C_{14}H_9NO^+$	0.24
610.27612	207.09168	$C_{14}H_{11}N_2^+$	0.04
610.29307	208.07575	$C_{14}H_{10}NO^+$	0.29
610.29219	208.09957	$C_{14}H_{12}N_2^+$	0.32
610.26145	209.07103	$C_{13}H_9N_2O^+$	0.41
610.32320	217.13349	$C_{13}H_{17}N_2O^+$	-0.24
610.29549	220.09954	$C_{15}H_{12}N_2^+$	0.20
610.29365	221.10735	$C_{15}H_{13}N_2^+$	0.10
610.31504	222.11525	$C_{15}H_{14}N_2^+$	0.46
610.29262	223.12304	$C_{15}H_{15}N_2^+$	0.28
610.28440	224.09451	$C_{14}H_{12}N_2O^+$	0.43
610.28418	225.10226	$C_{14}H_{13}N_2O^+$	0.09
610.25980	237.10229	$C_{15}H_{13}N_2O^+$	0.19
610.24685	243.11282	$C_{14}H_{15}N_2O_2^+$	0.07
610.27628	245.12846	$C_{14}H_{17}N_2O_2^+$	0.03
610.28918	251.11793	$C_{16}H_{15}N_2O^+$	0.15
610.27908	261.13865	$C_{18}H_{17}N_2^+$	0.11

610.30485	267.13656	$C_{16}H_{17}N_3O^+$	-0.19
610.29846	268.14446	$C_{16}H_{18}N_3O^+$	0.08
610.27960	277.13364	$C_{18}H_{17}N_2O^+$	0.36
610.24110	304.12069	$C_{19}H_{16}N_2O_2^+$	0.19
610.29122	305.12853	$C_{19}H_{17}N_2O_2^+$	0.23
610.28845	307.14415	$C_{19}H_{19}N_2O_2^+$	0.15
610.29899	315.17029	$C_{18}H_{23}N_2O_3^+$	-0.08
610.31223	319.16789	$C_{20}H_{21}N_3O^+$	-0.08
610.26915	320.17561	$C_{20}H_{22}N_3O^+$	-0.40
610.29246	325.15468	$C_{19}H_{21}N_2O_3^+$	0.02
610.25389	338.18615	$C_{20}H_{24}N_3O_2^+$	-0.46
610.25168	342.18157	$C_{19}H_{24}N_3O_3^+$	1.02
610.28900	346.15441	$C_{21}H_{20}N_3O_2^+$	-1.70
610.29349	347.16286	$C_{21}H_{21}N_3O_2^+$	0.09
610.29056	348.17071	$C_{21}H_{22}N_3O_2^+$	0.17
610.28948	488.18408	$C_{30}H_{24}N_4O_3^+$	-0.44

Average of absolute value of mass accuracy (fragment *m/z*): 0.32 ppm.

Precursor *m/z* range: 111 mDa (average precursor *m/z* 610.2871).

Precursor *m/z* standard deviation: 22 mDa.

Note : the precision of the precursor m/z should be 1 decimal place. The excessive number of decimals was chosen to highlight how far the reader should trust the data.
